# Genetic and phenotypic characterization of rice grain quality traits to define research strategies for improving rice milling, appearance, and cooking qualities in Latin America and the Caribbean

**DOI:** 10.1002/tpg2.20134

**Published:** 2021-09-12

**Authors:** Maribel Cruz, Juan David Arbelaez, Katherine Loaiza, Juan Cuasquer, Juan Rosas, Eduardo Graterol

**Affiliations:** ^1^ FLAR (Fondo Latinoamericano para Arroz de Riego) CIAT (International Center for Tropical Agriculture) Kilómetro 17 c, CP, Cali Valle del Cauca 763537 Colombia; ^2^ Dep. of Crop Sciences, Univ. of Illinois Urbana‐Champaign Turner Hall N‐211|1102 S. Goodwin Ave. | 046 Urbana IL 61801 USA; ^3^ CIAT (International Center for Tropical Agriculture) Kilómetro 17 Recta Cali, Palmira, CP, Cali Valle del Cauca 763537 Colombia; ^4^ INIA (Instituto Nacional de Investigación Agropecuaria) Ruta 8 Km. 281/33000 Treinta y Tres Uruguay

## Abstract

Rice (*Oryza sativa* L.)grain quality is a set of complex interrelated traits that include grain milling, appearance, cooking, and edible properties. As consumer preferences in Latin America and the Caribbean evolve, determining what traits best capture regional grain quality preferences is fundamental for breeding and cultivar release. In this study, a genome‐wide association study (GWAS), marker‐assisted selection (MAS), and genomic selection (GS) were evaluated to help guide the development of new breeding strategies for rice grain quality improvement. For this purpose, 284 rice lines representing over 20 yr of breeding in Latin America and the Caribbean were genotyped and phenotyped for 10 different traits including grain milling, appearance, cooking, and edible quality traits. Genetic correlations among the 10 traits ranged from −0.83 to 0.85. A GWAS identified 19 significant marker/trait combinations associated with eight grain quality traits. Four functional markers, three located in the *Waxy* and one in the *starch synthase IIa* genes, were significantly associated with six grain‐quality traits. These markers individually explained 51–75% of the phenotypic variance depending on the trait, clearly indicating their potential utility for MAS. Cross‐validation studies to evaluate predictive abilities of four different GS models for each of the 10 quality traits were conducted and predictive abilities ranged from 0.3 to 0.72. Overall, the machine learning model random forest had the highest predictive abilities and was especially effective for traits where large effect quantitative trait loci were identified. This study provides the foundation for deploying effective molecular breeding strategies for grain quality in Latin American rice breeding programs.

## Core Ideas

- Latin America rice germplasm exhibit complex genetic correlations among grain quality traits relevant for breeding.
- Amylose‐content, gelatinization temperature, and setback are ideal targets for marker‐assisted selection in Latin America *indica* germplasm.
- Random forest models displayed higher prediction abilities for oligogenic grain quality traits.

**Abbreviations**
1k‐RiCA: 1K‐Rice Custom Amplicon
AC: amylose‐content
BDv: breakdown‐viscosity
BL: Bayesian LASSO
BLUE: best linear unbiased estimate
CIAT: International Center for Tropical Agriculture
DHRR: delayed head rice recovery
FLAR: Latin American Fund for Irrigated Rice
GBSS: granule‐bound starch synthase
GL: grain length
GLWR: grain length to width ratio
GS: genomic selection
GT–ASV: gelatinization‐temperature expressed as alkali‐spread value
GW: grain width
HRR: head‐rice‐recovery
LAC: Latin America and the Caribbean
MAS: marker‐assisted selection
NIR: near‐infrared spectroscopy
OHRR: optimal head rice recovery
PCA: principal component analysis
QTL: quantitative trait loci
RF: random forest
RKHS: reproducing kernel Hilbert space
rrBLUP: ridge regression BLUP
RVA: Rapid Visco Analyzer
SBv: setback‐viscosity
SNP: single nucleotide polymorphism
SSIIastarch synthase IIaVIOFLAR: starch synthase IIaVIOFLARViveros de Observación
VIP: VIOFLAR Informative Panel
WCRK: white core rice kernel.

## 1 INTRODUCTION

Since 1948, rice (*Oryza sativa* L.) production spread and became a significant source of food and income in Latin America and the Caribbean (LAC) (Sanint, 1992). Rice breeding has made essential contributions in LAC, with more than 400 rice cultivars released from 1975 to 2012. These cultivars contributed to raising rice production in LAC to 27 million t of paddy grain grown in 5.7 million ha across the region (Martínez et al., 2014). The Latin American Fund for Irrigated Rice or FLAR is a public–private partnership that generates and disseminates knowledge, technologies, and innovations, contributing to rice competitiveness and sustainability in LAC. Since 1995, FLAR's breeding program has been developing improved rice germplasm and distributing it across different rice institutions in 17 countries in LAC. Elite FLAR's germplasm with high yield, disease resistance, abiotic tolerance, desired grain appearance, cooking, and milling quality are released each year in nurseries known as Viveros de Observación FLAR or VIOFLAR. Until this date, 87 rice cultivars, released in 14 different countries, were derived from VIOFLAR lines.

Grain quality is a significant breeding target in FLAR's breeding program. Consumer preferences such as grain color, shape, size, uniformity, softness, and looseness are inherent attributes that acquire meaning through the historical, geographical, and sociocultural context in which rice consumption is rooted (Custodio et al., 2019). As rice markets become increasingly complex, breeding programs must prioritize quality to develop acceptable cultivars for consumers (Calignacion et al., 2014; Custodio et al., 2019).

The Grain Quality Lab at FLAR has developed and implemented methodologies to characterize rice grain quality based on three main components: milling efficiency, shape and appearance, and cooking and edible qualities. Milling efficiency determines the final whole‐grain yield and the milled rice's broken kernel rate, which concerns the milling industry, consumers, and farmers. Head‐rice‐recovery (HRR), defined as the percentage of whole milled grain to paddy rice, is one of the parameters used to evaluate the milling process's quality and one of the factors determining rice market value (Nelson et al., 2011). Genetically, rice milling quality is controlled by the maternal diploid genotype, triploid endosperm genotype, and cytoplasmatic genomes (Zheng et al., 2007). Additive, epistatic, environmental, and environment × genetics interactions influence rice milling quality (Nelson et al., 2011; Tan et al., 2001; Zheng et al., 2007). A previous study using 355 *indica* elite lines from the International Rice Research Institute showed that HRR is negatively correlated with grain length and positively correlated with grain width (GW) (Zhou et al., 2015). Short and broad grains have higher HRR than long and slender grains (Zhou et al., 2015). In addition, this study also found that high percentage of grains with chalkiness was significantly (*P* value <.05) negatively correlated with HRR suggesting that HRR decreases rapidly with increasing percentage of grains with chalkiness and grain length suggesting that is possible to select for high HRR indirectly using these two traits (Zhou et al., 2015).

Grain shape and size defined by the grain‐length and ‐width millimeters are prime breeding targets as they affect yield and quality (Wang et al., 2012). Slender grains classified as having a length‐to‐width ratio of three and above are preferred by the majority of consumers (Fan et al., 2006; Jain et al., 2004). Multiple genes have been shown to control grain size; among them, *GS3* and *DEP1* were associated with grain length (Huang et al., 2009; Mao et al., 2010), and *GW2*, *qSW5*, and *GS5* regulate grain width (Li et al., 2011; Shomura et al., 2008). Grain appearance is a crucial property affecting its market acceptability. Rice cultivars with translucent white endosperm are preferred in most rice markets (Lin et al., 2016). Chalkiness, characterized by opaque portions found in rice endosperm caused by the loose starch granules, is an undesirable trait that negatively affects appearance and milling quality (Li et al., 2014). When chalkiness located in the grain's central area is known as white‐core rice kernel (WCRK) (Zhang et al., 2014). High WCRK results in more broken polished rice and worse taste (Peng et al., 2014). White‐core rice kernel occurs when environmental conditions are unfavorable or poor translocation of assimilates from the source leaves and stems at the early and middle stages of grain filling (Zhang et al., 2014). This type of chalkiness is a complex quantitative trait controlled by many genes and influenced by environmental factors such as temperature, humidity, and field fertility (Li et al., 2014). Genetically, the loci *Chalk5* (Li et al., 2014), *qPGWC‐7* (Zhou et al., 2009), *qPGWC‐8* (Gou et al., 2011), *qACE‐9* (Gao et al., 2016), and *qPCG1* (Zhu et al., 2018) have been associated with grain chalkiness.

Rice physicochemical properties impact cooking and edible qualities. Amylose‐content (AC) and gelatinization‐temperature are the most widely used determinants for rice texture and processing properties (Chen et al., 2008). Amylose‐content is one of the significant traits used in the selection process for eating quality among rice breeding programs. It influences texture and cooked grains' potential to retrograde after cooking (Calingacion et al., 2014) and AC can be used to classify rice grain quality into waxy (0–2%) and nonwaxy types, with nonwaxy subclassified into low (12–20%), intermediate (20–25%), and high (25–33%) AC types (Lu et al., 2009). The *Waxy* (*Wx*) gene, located on chromosome 6, encodes the enzyme granule‐bound starch synthase (GBSS) required for the synthesis of amylose in the endosperm (Chen et al., 2008). Two *Waxy* gene alleles, *Wx^a^* and *Wx^b^*, have traditionally been associated with the content of GBSS transcript and amylose content in rice endosperm, with the *Wx^a^* allele synthesizing higher contents of GBSS and thus exhibiting higher amylose‐content than the *Wx^b^* allele (Chen et al., 2008). In addition to the significant effect of the *Waxy* gene, minor genes and the environment were also thought to influence rice amylose content (Bao et al., 2004).

Gelatinization‐temperature is the temperature that melts the crystalline portion of the amylopectin structure. This starch characteristic is associated with the proportion of amylopectin short A and B1 chains, which is primarily controlled by the gene *starch synthase IIa* (*SSIIa*) (Umemoto et al., 2004; Umemoto & Aoki, 2005). Rice grains with low, intermediate, and high gelatinization‐temperatures show complete, partial, and no disintegration when treated with alkali solution and assessed for extending of dispersal (Pang et al., 2016). The degree of spreading value of individual milled grains in a weak alkali solution of 1.7% potassium hydroxide (w/v; KOH) is highly correlated with gelatinization‐temperature (Graham, 2002). Rice with low gelatinization‐temperature easily disintegrates under KOH solution showing high alkali‐spread values. Samples with intermediate and high gelatinization‐temperature showed partial and no disintegration in KOH showing intermediate and low alkali‐spread values, respectively (Graham, 2002). Gelatinization‐temperature is positively correlated with the amount of time required to cook rice (Waters et al., 2006). Rice cultivars with high gelatinization‐temperature require more water and cooking time than those with low or intermediate gelatinization‐temperatures. A low‐to‐intermediate gelatinization‐temperature is desired for high‐quality rice cultivars (Pang et al., 2016).

**Core Ideas**

- Latin America rice germplasm exhibit complex genetic correlations among grain quality traits relevant for breeding.
- Amylose‐content, gelatinization temperature, and setback are ideal targets for marker‐assisted selection in Latin America *indica* germplasm.
- Random forest models displayed higher prediction abilities for oligogenic grain quality traits.

The variables AC and gelatinization‐temperature alone do not explain all the variation for eating cooking quality. The pasting properties of starch measured with a Rapid Visco Analyzer (RVA) are also important factors affecting eating and cooking quality (Balet et al., 2019; Pang et al., 2016). Rice viscosity profiles can establish the gelatinization capacity of starch and texture, which increases grain rigidity after cooking (Cozzolino et al., 2016; Yan et al., 2005). The grain quality variables breakdown‐viscosity (BDv) and setback‐viscosity (SBv) are rheological properties measured with RVA, associated with starch softness and cooking quality. The variable BDv defines the water absorption capacity during cooking. Setback‐viscosity represents the molecular restructuring of starch according to the equilibrium between free and bind water molecules at the end of cooking (Bao et al., 2000; Pang et al., 2016). Rice texture and looseness were strongly correlated with BDv and SBv (Pang et al., 2016). A genome‐wide association study (GWAS) performed in a diversity panel of *indica* rice detected six QTLs in chromosome 6, 7 and 11 associated with BDv and SBv (Xu et al., 2016).

The Latin American Fund for Irrigated Rice has defined specific breeding targets for LAC to develop germplasm with HRR (≥60%), AC (≥26.5%), gelatinization‐temperature expressed as alkali‐spread value (GT–ASV ≥5), BDv measured in viscosity units (mPa/s) (≤800), SBv measured in viscosity units (mPa/s) (1,600), the grain‐length‐to‐width ratio in millimeters (GLWR ≥3.2 mm), and WCRK (≤7.08%). Until this date, breeding for different grain quality traits has relied on phenotypic selection, with quality traits measured at different breeding‐cycle stages. Advancements in the sequencing and genotyping technologies have led to the development of next‐generation sequencing platforms that allow the cost‐effective implementation of high‐throughput genotyping for breeding applications (Hickey et al., 2019). Molecular‐based breeding methods such as marker‐assisted selection (MAS), marker‐assisted‐backcrossing (MABC), and genomic selection (GS) offer the opportunity to increase breeding efficiency, selection intensity, and decrease breeding cycles for grain quality (Crossa et al., 2017).

In this study, a set of VIOFLAR elite lines that represent the genetic diversity and breeding history from 1995 to 2015 of FLAR's breeding program (VIOFLAR Informative Panel [VIP]) was genetically and phenotypically characterized for grain milling efficiency (HRR at optimal [OHRR]and delayed [DHRR] harvest), shape and appearance (GW, grain length [GL], GLWR, and WCRK), and cooking and edible qualities (AC, gelatinization‐temperature, BDv, and SBv) to (a) identify genetic factors associated with grain quality traits, (b) determine entry points to implement marker‐based assisted selection strategies for grain quality, and (c) determine genetic trends for grain quality traits during the past 20 yr in FLAR's tropical breeding program.

## 2 MATERIALS AND METHODS

### 2.1 Plant materials

A sample of 284 rice lines representing the diversity of different VIOFLAR's nurseries spanning 17 yr from 1999 to 2015 was genotypically and phenotypically characterized for grain quality in this study. These lines were selected using a multivariate clustering analysis on phenotypic information collected on 3,400 lines from 17 VIOFLAR nurseries from 1999 to 2015. The phenotypic information used to choose the lines included early‐vigor, flowering‐time, blast, and *Rice hoja blanca virus* resistance, WCRK, AC, HRR, and yield. Once clusters were defined, a balanced number of lines from each VIOFLAR/year nursery were randomly sampled from each group to integrate the VIP set. This approach minimized bias during the selection of lines and improved the selection of germplasm representing the breeding history of FLAR. Eleven checks and donor lines routinely used in FLAR's tropical breeding program were included in the experiments. Detailed information on the germplasm used in this study can be found in Supplemental File 1 “VIP accessions info.”

### 2.2 Grain quality phenotypic evaluation

The set of 284 VIP lines and 11 checks were evaluated at two experimental stations located in the headquarters of the International Center for Tropical Agriculture (CIAT) in Colombia: Palmira and the Santa Rosa Experimental Station at Villavicencio. Detailed information on the Experimental Stations can be found in the Supplemental File 1‐“Experimental Stations info.” Rice trials at Palmira were grown during two seasons, in the first and second halves of 2016, under full‐irrigated conditions with rows established through transplantation using five plants or hills per linear meter. Historically, Palmira has been used as the optimal site to evaluate rice lines’ yield potential, milling, and cooking quality. Trials conducted in Santa Rosa were planted in the first halves of 2016 and 2017 under upland conditions with rows established using direct seeding. Santa Rosa is a strategic hot‐spot or ideal site to evaluate disease resistance and abiotic stresses for rice production and WCRK due to its high humidity and temperature. Trials in Palmira were used to measure the quality traits at OHRR and DHRR dates, AC, GT–ASV, BDv, SBv, GL, GW, and GLWR. Trials conducted in Santa Rosa were used to measured WCRK.

Rice lines in all trials were evaluated using an Alpha Lattice experimental design (Paterson et al., 1978) with three replications. In Palmira, plots had three rows spaced 0.3 m from each other with 10 hills on each row spaced 0.2 m, for a total plot area of 2 m^2^. Grains from each plot were harvested between 20 and 24% moisture content from eight hills located in the mid‐row to avoid border effect. Harvested paddy grains were dehulled and polished using a small Satake rice mill (https://satake‐group.com/index.html). Milled‐grain harvested at Palmira was used to quantified OHHR, DHRR, AC, GT–ASV, BDv, SBv, GL, GW, and GLWR. Milled rice harvested at Santa Rosa was used to measured WCRK.

The traits GW, GL, GLWR, and WCRK were determined using milled grain images and the software ImageJ (http://rsb.info.nih.gov/ij/). Photographs were taken on 1.3 g of milled‐whole grains over a contrasting background and homogeneous light conditions (Santos et al., 2019). Grain images were then processed using ImageJ that measured individual GL on the *x*‐axis and GW on the *y*‐axis (Santos et al., 2019). Each measurement was recorded and then averaged to generate the sample GL and GW values scaled in millimeters. The composed trait GLWR was calculated by dividing the average GL by GW (GLWR = GL/GW). The grain appearance quality trait WCRK is the presence of nontranslucent areas in the center of the grain endosperm. To calculate WCRK, two parameters were estimated from milled grain images: the total‐grain‐area and the grain area that is translucent (or total‐translucent‐area). The WCRK was calculated as the percentage of the total grain area that is not translucent as WCRK = 1 – TTA/TGA. Grain area was estimated by transforming the image channel to hue, saturation, and brightness, then binarized to black and white, and black pixels recorded and scaled in square millimeter based on a calibration image.

The apparent AC in grain was evaluated using a near‐infrared spectroscopy (NIR) protocol. Samples were prepared by drying them to 13% moisture content with an SKS‐480 Grain Dryer (Suncue Company, https://suncue.en.taiwantrade.com/). About 6 g of milled rice from each sample was grounded using a Cyclotec CT 293 mill (https://www.fishersci.com/shop/products/ct‐293‐cyclotec‐115v‐60hz/12053116) to obtain powder particle sizes less than 0.05 mm. The NIR system FOSS NIR 6500 (https://www.fossanalytics.com/en) was used to measure AC. The equipment was calibrated according to apparent AC method AACC Method 61‐03.01 (http://methods.aaccnet.org/summaries/61‐03‐01.aspx). The NIR chemometric model was developed using 1,793 rice samples ranging from 13 to 33% AC. The calibration curve was built by regressing AC values determined using the potassium iodine colorimetric protocol (Graham, 2002) with the NIR reference values scanned in rice flour. The final NIR model for AC was described as *Y* = 0.9732, *X* – 0.00015 and showed a coefficient of correlation (RSQ) = 0.97, standard error of calibration (SEC) = 0.95, standard error of cross validation (SECV) = 0.96, coefficient of determination (1 – VR) = 0.94, and standard deviation (SD) = 6.3. Finally, the standard error prediction of the calibration model (SEP) was estimated to be 1.57. This methodology has been routinely used to estimate AC in FLAR's Grain Quality Lab (https://flar.org/en/). For each sample, 5 g of flour was loaded in a circular sample cup and pressed slightly to obtain a similar packing density. Sample spectra were collected continuously over a wavelength range between 400 and 2,500 nm using the software ISIScan. Spectra data were converted into a percentage of AC in rice starch for each sample.

The rice starch quality trait GT was measured using the proxy methodology by alkali spreading or digestion test (Graham, 2002). The degree of spreading value (GT–ASV) of individual milled rice kernels in a weak alkali solution (1.7% KOH; w/v) is highly correlated with GT. Rice with low GT disintegrates completely, showing higher spreading values, whereas rice with an intermediate GT shows partial disintegration and rice with high GT remains largely unaffected in the alkali solution with low spreading values. A duplicate set of six whole‐milled kernels were selected and treated with 10 ml of 1.7% KOH solution (w/v). The samples were then arranged to provide enough space between kernels to allow for spreading. Samples were incubated for 23 h in a 30 °C oven. Treated rice samples are scored visually using a numerical scale from 1 (high GT) to 7 (low GT). Standard low, intermediate, and high GT checks cultivars are included in every test (Graham, 2002). In this study, GT is from now on expressed in alkali‐spread values or GT–ASV.

The pasting properties BDv and SBv of starch were measured using a Perten's RVA 4500‐instrument (https://www.perkinelmer.com/corporate/perten). For each analyzed sample, 3 g of rice flour was diluted in 25 g of distilled water. The AACC Method 61‐02.01 was used to determined gelatinization and paste viscosity properties in rice flour (ICC, 2004; http://methods.aaccnet.org/summaries/61‐02‐01.aspx). Each sample was stirred at 960 and 160 rpm for 10 s and 14 min, respectively. The heating cycle began at 50 °C for one minute, followed by 95 °C for 7 min, and ended at 50 °C for 4 min. Rapid viscosity analyzer curves were registered, and the values for BDv and SDv of starch measured in Rapid Viscosity Units (mPa/s) were extracted from curves using Thermocline for Windows v11.2 software (https://www.perkinelmer.com/corporate/perten).

The milling quality traits OHHR and DHRR were determined using the protocol developed by Berrio et al. (2002). First, rice paddy from each sample was harvested at around 18% moisture content for each genotype and was dried down to 13%. Two subsamples were taken from dried paddy rice to measure OHHR and DHRR, respectively. For OHHR, 100 g of paddy rice was dehulled and polished using a small Satake rice mill (https://satake‐group.com/index.html) and McGill #2 Mill polisher (Rapsco). Whole grains were separated using the rice milled and weighted in grams (g). The percentage in weight of whole grains recovered from the original paddy sample was used to determine the HRR values.

To determine DHHR, delayed harvesting conditions were recreated using a modified tub that contains 72 samples, water, and airflow compartments that simulate field conditions where the grains undergo rewetting after optimal drying (Berrio et al., 2002). In each simulation batch, 72 samples of 115 g of paddy rice were placed in individual sample chambers. The samples were then submerged in 0.4 m^3^ of water at a constant temperature of 25 °C for 2 h. Water was drained, and the air was pumped with a fan for 18.75 h at a temperature of 26 °C. After this treatment, the air temperature was raised using a heating system to 29 °C for 2.25 h to decrease samples moisture‐content to 12%. Samples were then transferred into paper envelopes and stored at room temperature for 10–12 d. After this time, 100 g of each sample was used to determine head rice recovery values and expressed as DHHR. The acronyms describing each variable and raw phenotypic values for each variable can be found in Supplemental File 1 “Phenotyping info” and “Phenotypic raw data.”

### 2.3 Genotyping and SNP calling, filtering, and formatting

The VIP lines and checks used in this study were genotyped using the 1K‐Rice Custom Amplicon (1k‐RiCA assay), as described in Arbelaez et al. (2019). Genomic DNA was obtained from leaf tissue of single plants collected at CIAT, and DNA extraction, genotyping, and single nucleotide polymorphism (SNP) calling was done using the genotyping services of AgriPlex GENOMICS (https://agriplexgenomics.com/) at Cleveland, OH. Their proprietary platform PlexSeq was adapted to create the 1k‐RiCA.v2. A custom SNP‐calling pipeline developed by AgriPlex GENOMICS was used to assign variants on the 1k‐RiCA amplicons through alignment to the Nipponbare rice genome MSU7 version (Kawahara et al., 2013).

Single nucleotide polymorphisms with minor allele frequency ≤0.01; heterozygous calls ≥10%; and call rate ≤75% were removed using custom scripts written in R version 3.5.0 (R Core Development Team, 2018) and deposited in Github (https://github.com/jdavelez/1k‐RiCA‐geno‐filters/blob/master/jdavelez_1k‐RiCA.R). For each SNP, heterozygosity was determined as the proportion of heterozygous calls among all successfully called genotypes. The SNP call rate was defined as the proportion of successfully called genotypes among all samples used in the study. Final SNP data were merged and formatted in a single data frame with markers in rows and samples in columns. The SNP identification, chromosome, and physical position information are given in the data frame's first three columns. The genotypic data frame generated in this study is available in Supplemental File 1 “Genotypic raw data.”

To prepare the marker data for GWAS, GS, and population‐structure analyses, the genotypic data set coded as dinucleotide genotypes was formatted to numerical genotypes reflecting allele dosage either as 0, 1, and 2, or −1, 0, and 1 using the R function ‘*geno_to_allelecnt*’ deposited in the GitHub site developed by Dr. Eva Chan (https://github.com/ekfchan/evachan.org‐Rscripts).

### 2.4 Population structure

To assess the VIP set population structure, the numerical genotypes coded as −1, 0, and 1 from all samples were used to estimate an additive relationship matrix (**K**) (Endelman et al., 2011) using the ‘*A.mat*’ function from the R package ‘*rrBLUP*’ (Endelman et al., 2011). A principal component analysis (PCA) (Pearson, 1901) was performed on the **K** matrix using the R function ‘*prcomp*’ (Mardia et al., 1979; R Core Team, 2018). Principal components were visualized using the R package ‘*ggplot2*’ (Wickham, 2009).

An analysis to estimate the degree of variation between the VIOFLAR nurseries was assessed using pairwise *F*_ST_ values calculated between VIOFLAR groups using the function ‘*calc_wcFst_spop_pairs.R*’ developed by Chan (2014) (http://evachan.org) that estimate *F*_ST_ (theta) values for each pair of VIOFLAR nurseries, using the method described by Weir and Cockerham (1984). A Neighbor‐Joining analysis using Saitou and Nei (1987) tree estimation was used to visualize the results.

### 2.5 Phenotypic data analysis

#### 2.5.1 Broad sense heritability

Analyses of variance (ANOVAs) were conducted for all‐grain quality traits by using the R package ‘*asreml*’ (ASReml‐R Version 4 ASReml; Butler et al., 2018) with the following statistical model: *Y_i,j,k_* = μ = *g_i_* + β*_j_* + *s_j_*_(_ *_i_*_)_ + *r_k_*_(_ *_i,j_*_)_ + ε*_i,j,k_*, where *Y_i,j,k_* is the phenotype observations, μ is the overall mean, *g_i_* is the effect of a genotype considered random with a normal distributions *N*(0, σ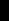) without considering the additive relationship matrix as the variance–covariance matrix, and *i* varying from 1 to 284 for 284 genotypes, β*_j_* was the fixed effect of season, with *j* ranging from 1 to 2 for two different seasons, *s_j_*_(_ *_i_*_)_ was the random effect of the genotype by season interaction effect with a normal distribution *N*(0, σ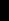), *r_k_*_(_ *_i,j_*_)_ was the random effect of the replication (or block) within a season for each genotype, with *k* ranging from 1 to 3 for three different replications and a normal distribution *N*(0, σ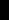), ε*_i,j,k_* was the residual considered as random and following a normal distribution *N*(0, σ^2^). Broad sense heritability of accession means, *H* ^2^, was calculated for each trait using the formula of Hallauer et al. (2010) as follows:

$$H^{2}=\frac{\sigma_{g}^{2}}{\sigma_{g}^{2}+\frac{\sigma_{gs}^{2}}{j}+\frac{\sigma_{e}^{2}}{jk}}$$

where *j* represents the mean number of seasons in which accessions were tested, and *k* represents the mean number of plots for each accession within the season. Genotypes were assumed independent and identical distributed for estimating *H* ^2^. Variance components were calculated from the previously described linear mixed model using ‘*asreml*’ using the residual maximum likelihood estimation or RMEL method.

#### 2.5.2 BLUEs estimation and phenotypic clustering analysis for grain quality and grain appearance properties

A clustering analysis on the VIP set based on the grain quality traits OHHR, AC, GT–ASV, BDv, SDv, GLWR, and WCRK was done using the best linear unbiased estimate (BLUE) effects calculated OHHR, AC, GT–ASV, BDv, SDv, GLWR, and WCRK traits on each VIP line. The BLUE values were estimated using the same model described to calculated broad‐sense heritability; however instead of treating genotypes as random, they were treated as a fixed effect. Calculated BLUE vectors for each trait were scaled, centered, and used to create a matrix for clustering. The optimal number of clusters was determined using the Gap Statistic method described by Tibshirani et al. (2001). The Gap Statistic was applied to the K‐means clustering method performed on the scaled phenotypic matrix using Hartigan and Wong (1979) K‐means clustering algorithm. The R function ‘*clusGap*’ from the package ‘*factoextra*’ (Kassambara, Mundt, & Kassambara, 2017) was used to compute the gap statistics with K‐means as the clustering function with 1,000 Monte Carlo bootstrap sampling set in the function parameters (https://afit‐r.github.io/kmeans_clustering). The optimal cluster number was used to group the VIP lines. The results from this analysis and phenotypic PCA were visualized using the R package ‘*ggplot2*’ (Wickham, 2009) and ‘*ggrad*’ (Ricardo Bion, https://github.com/ricardo‐bion/ggradar).

#### 2.5.3 Genetic correlation between grain quality traits

Genetic correlations between grain quality traits were estimated using a multivariate mixed model analysis in ‘*asreml*’ (ASReml‐R Version 4 ASReml; Butler et al., 2018). Each multivariate mixed model was defined in the function ‘*asreml()*’ by specifying a matrix of traits as the fixed effects formula's response. Internally ‘*asreml*’ expands the data frame by repeating each row by the number of traits such as the traits are nested within each experimental unit as described by ASReml‐R‐Reference‐Manual‐4 (Butler et al., 2018). The model y=Xβ+Zu+e, where 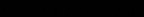, 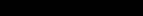, and 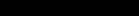, and y is the response vector of **d** traits (grain quality traits described above), **X** is a design matrix for fixed effects β that included the grand mean and the trial‐season for each trait, and **Z** is a design matrix for random genetic effects **u**. Following a multivariate normal distribution $\left(N_{m}\right)$, the marginal density of y is $\left(y\beta ,R,G\right)∼N_{m}\left(X\beta ,V\right),$ and $V\;=\;Z\left(G⊗K\right)Z^{T}+\left(R⊗I\right)$. The matrices G and R are dxd symmetric unstructured genomic error covariance matrices respectively, I is an identity matrix, K remains the additive genomic relationship matrix calculated using the numerical genotype matrix for the VIP lines and the function ‘*A.mat*’ from the R package ‘*rrBLUP*’ (Endelman et al., 2011), and e is the residual with $e\;∼\;N(0,{\;\sigma }_{e}^{2}I)$. The error structure was specified as two‐dimensional, with independent units and unstructured variance. The results from each analysis were visualized using the R package ‘*ggplot2*’ (Wickham, 2009).

#### 2.5.4 Genome‐wide association studies for grain appearance, quality, and milling traits

A univariate linear mixed model GWAS that accounts for spatial variation across and within the season for each trait was fit using the R Package ‘*sommer*’ (Covarrubias‐Pazaran, 2016). To account for spatial variation across and within seasons, the following model was used: y=Xβ+Zu+Wτ+e, where y is the response vector of a trait, β is the vector of fixed effects with the design matrix X (relating observations to fixed effects, in this case, the grand mean and the trial‐season for each trait; **u** is a vector of random effects with the design matrix Z (relating the genotypes, the nested effect of rep‐within‐trial‐season, and the interaction between genotypes and season) with $u∼N(0,\sigma_{u}^{2}K)$ and K as the genomic relationship matrix; the variable τ models the additive SNP effect as a fixed effect; e is the residual effect vector assumed to be normally distributed with $e∼N(0,\sigma_{u}^{2}I)$.

Because of VIP lines’ low subpopulation structure, a model accounting for familial relatedness (K) with genotype as random effect structured following a kinship matrix K. The inspections of the quantile–quantile (*Q–Q*) plots of the respective P‐values obtained in each model were used to assess to what extend the models accounted for genetic relatedness among the VIP lines and therefore guarded against false discoveries. The observed P values (on a *−log_10_* scale) of each SNP were plotted against their chromosomal position to produce Manhattan plots using the R package ‘*qqman*’ (Turner et al., 2018). A threshold of *−log_10_* (P‐value) = 4.23 estimated using the Bonferroni multiple testing correction method with a statistical significance value of .05 [$-log_{10}\;\left(\frac{0.05}{863\;\mathrm{SNPs}}\right)=\;4.23]$ (Bonferroni, 1935), was used to define the significant association between SNP and trait phenotypes. After each GWAS, significant SNP associated with each grain quality traits were subset and the marker name, chromosome, physical position, additive marker effect, *−log_10_* (P‐value), the proportion of genetic variance explained by the marker were extracted and summarized as part of the results.

Marker haplotypes analyses were performed for AC, GT–ASV, and SB_V_. Haplotypes defined using markers associated with each trait were regressed against the adjusted means calculated for each accession. Multiple comparisons among haplotypes were estimated using Tukey's HSD (honestly significant difference) (Brillinger & Tukey, 2002) method using the R function ‘*HSD.test*’ from the R package ‘*agricolae*’ (Mendiburu 2015). Significant differences between groups were determined by different letters in boxplots.

#### 2.5.5 GS for grain milling, appearance, and cooking quality traits

Four different GS models were used to calculate genomic estimated breeding values to test each model's predictive ability effect. The GS models tested included ridge regression (rrBLUP) (Endelman et al., 2011), Bayesian LASSO (BL) (Park & Casella, 2008), reproducing kernel Hilbert space regressions (RKHS) (de los Campos et al., 2009), and random forest (RF) (Breiman, 2001). These models were specifically selected to represent a range of different assumptions on QTL effect distributions resulting in different marker effect distributions (Heslot et al., 2012). The rrBLUP model assumes that marker effects are homogeneously distributed across the loci, whereas BL allows heterogeneity among markers, with some markers having larger effects than others. Nonparametric models such as RF, a machine learning‐based method, and RKHS consider correlations or interactions between markers, capturing nonadditive genetic effects.

For rrBLUP, the marker‐based genomic relationship matrix K was constructed using numerically coded genotypes and the ‘*A.mat*’ function in the ‘*rrBLUP*’ package (Endelman, 2011). The ridge regression model was tested using the R package ‘*rrBLUP*’ (Endelman et al., 2011) by implementing the mixed model y=Xβ+Zu+e, where yis a vector of phenotype observations, βis a vector of fixed effects for the seasons and X is the design matrix for seasons, uis a vector of random additive marker effects with $\mathrm{Var}\;\left[u\right]=\;K\sigma^{2}$, and e is a vector of residual effects with $\mathrm{Var}\;\left[e\right]=\;I\sigma_{e}^{2}$. To use RF, the genotype matrix was coded as 0, 1, and 2 to be consistent with the genotypic data format requirements of the ‘RandomForest’ R package. RF is a collection of classification trees grown on bootstrap samples of observations using a random subset of predictors to define the best split at each node. This model was implemented using the R package ‘*RandomForest*’ (Liaw & Wiener, 2003) using the default settings of the function except for the number of trees, which was set to 1000, and the minimum size of the terminal node as 50, as suggested by Heslot et al. (2012). The Bayesian model BL was implemented using the R package ‘*BGLR*’ (Pérez et al., 2014) by fitting the mixed model y=Xβ+Zu+e, as defined for rrBLUP. In this case the prior distribution of u is not considered normally distributed, and uis the corresponding vector of marker effects assigned IID double‐exponential priors $\mathrm{Pr}\left(u_{i}\lambda \right)=\;\frac{\lambda }{2}\exp\left(-\lambda |u_{i}|\right)$, which corresponds to the prior used in the *BL* model (Pérez et al., 2014). Additionally, the default parameters for thinning, 5, and 8,000 iterations with the first 1,000 iterations discarded as burn‐in were used as described by Pérez et al. (2014). The model *RKHS* (Gianola et al., 2008) implements the genomic relationship matrix used in *rrBLUP* and replaces it with a kernel matrix, which enables non‐linear regression in a higher‐dimensional feature space. According to Pérez et al. (2014) the *RKHS* model can be represented as y=1μ+u+ε, with $p\left(\mu ,u,\;\varepsilon \right)\propto N\left(u0,K\sigma_{u}^{2}\right)N\left(\varepsilon |0,\;I\sigma_{\varepsilon }^{2}\right)$, where $K\;=\;\{K(x_{i1},x_{i^{\prime }})\}$ is an (n×n)‐reproducing‐kernel‐matrix whose entries are functions of marker genotypes of pair of individuals from the relationship matrix, and ε is a vector of residuals on length n.

To compute predictive ability, a fivefold cross‐validation experiment using 4/5 of the 284 VIP lines as the training set to predict the remaining 1/5 VIP lines of the validation set was implemented. Each cross‐validation was repeated five times using five independent partitioning of the accessions into the training set and validation set. The presence of highly related individuals within each VIOFLAR nursery in the dataset could artificially inflate predictive abilities if the closest individuals were randomly assigned to the same fold and used a training‐set. A stratified cross‐validation strategy (Zeng & Martinez, 2000) was used to control for predictive abilities bias when designing the different folds by sampling individuals randomly within VIOFLAR nurseries defined using the VIP line information. Each cross‐validation experiment's prediction ability was computed as the mean value of the five Pearson correlations (Pearson, 1901) between the observations and the cross‐validated genomic estimated breeding values (Heslot et al., 2012).

#### 2.5.6 Grain quality genetic trends

The phenotypic and genotypic data collected in this study were used to determine the total and additive genetic trends observed across 17 yr of breeding history for the grain quality traits. Total and additive rate of genetic realized gain for individual traits was measured using the mean phenotypic and breeding values for the VIP and the VIOFLAR year (Rutkoski et al., 2019). The estimate of realized gain per year was quantified as the slope of the regression line of the phenotypic or breeding value on year number (Garrick, 2010). The total genetic trend for each trait was estimated by fitting the mixed model $y_{i,j,k,l}=\;\mu +\beta_{i}+g_{j}+s_{k(j)}+r_{l(j,k)}+\varepsilon_{i,j,k,l}$, in which $y_{i,j,k,l}$ is the phenotype, μ is the overall mean, $\beta_{i}$ is the fixed effect of the year when that VIP line was first tested in VIOFLAR trials (year of release), with j varying from 1999 to 2015 for 17 different VIOFLAR testing years, $g_{j}$ is the effect of a genotype considered random with a normal distributions $N(0,\;I\sigma_{G}^{2})$ without considering the additive relationship matrix as the variance–covariance matrix, and j varying from 1 to 284 for 284 genotypes, $s_{k(j)}$ is the random effect of the genotype × season interaction effect with a normal distribution $N(0,\;\sigma_{S}^{2})$, $r_{l(j,k)}$ is the random effect of the replication (or block) within a season for each genotype, and a normal distribution $N(0,\;\sigma_{R}^{2})$, and $\varepsilon_{i,j,k,l}$ is the residual considered as random and following a normal distribution $N(0,\;\sigma^{2})$. To estimate the additive genetic trend, the same model was fit considering the additive relationship matrix for the genotype random effect $g_{j}$ with a normal distribution $N(0,\;K\sigma_{G}^{2})$ in which K is the additive relationship matrix estimated using ‘*rrBLUP*’ (Endelman, 2011). A Wald test (Wald, 1943) was used to determine if the release or the VIP year was significant. Regressed adjusted mean breeding values for each year and trait were visualized using the R package ‘*ggplot2*’ (Wickham, 2009).

## 3 RESULTS

### 3.1 VIOFLAR Informative Panel genetic structure

**FIGURE 1:**
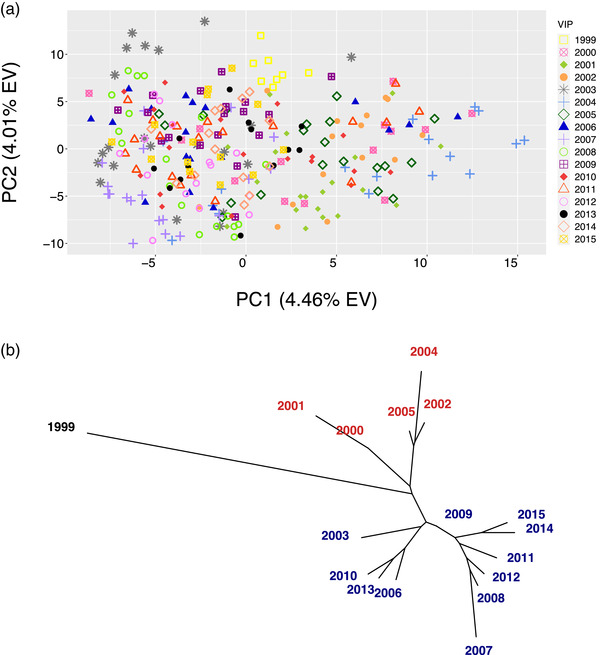
VIOFLAR Informative Panel (VIP) genetic structure. (A) Principal component analysis of 284 VIP lines from the 1999–2015 yr‐nurseries using the genotypic data generated with the 1k‐RiCA single nucleotide polymorphism (SNP) assay on each line. Scatter plot of PC1 vs. PC2. (B) Weir and Cockerham's *F*_ST_ Neighbor‐Joining analysis between the VIOFLAR's nurseries from 1999 to 2015 using the genotypic information of each line generated using the 1k‐RiCA SNP assay. Visually determined branches were color coded black, red, and blue, respectively

A PCA using the genotypic data of 284 VIP lines from 17 different VIOFLAR nurseries from 1999 to 2015 showed no major genetic differentiation among the VIP lines (Figure 1A and Supplemental Figure S1A and S1B). Together, the principal components (PC) 1, 2, and 3 explained 11.93% of the total genetic variation with PC1, PC2, and PC3, explaining 4.46, 4.01, and 3.46%, respectively (Figure 1A and Supplemental Figure S1A and S1B). An assessment to determine finer levels of genetic divergence between VIOFLAR nurseries was done using a pairwise *F*_ST_ analysis using the genotypic information collected in VIP lines grouped by each VIOFLAR nursery (Figure 1B, Supplemental Table S1). Pairwise *F*_ST_ values between VIOFLARs ranged from 0.02 to 0.26, with a mean of 0.081 (Supplemental Table S1). The *F*_ST_ results were visualized using an unrooted Neighbor‐Joining tree graph (Figure 1B). Three distinct groups with different genetic divergence levels were observed in the Neighbor‐Joining tree. The first group was made up of the 1999 VIOFLAR nursery (Figure 1B). The 1999 VIOFLAR showed average *F*_ST_ values of 0.185 and 0.193 concerning Groups 2 and 3, being the most divergent groups. The second group contained VIOFLAR nurseries from the Years 2000, 2001, 2002, 2004, and 2005 (Figure 1B), and the third group had VIOFLARs from the Years 2006 to 2015, including the 2003 nursery (Figure 1B). The average *F*_ST_ value observed between Groups 2 and 3 was 0.08 (Figure 1B and Supplemental Table S1). Groups 2 and 3 were more similar to each other when compared with Group 1.

### 3.2 Broad sense heritability and genetic correlations between grain quality traits

**FIGURE 2:**
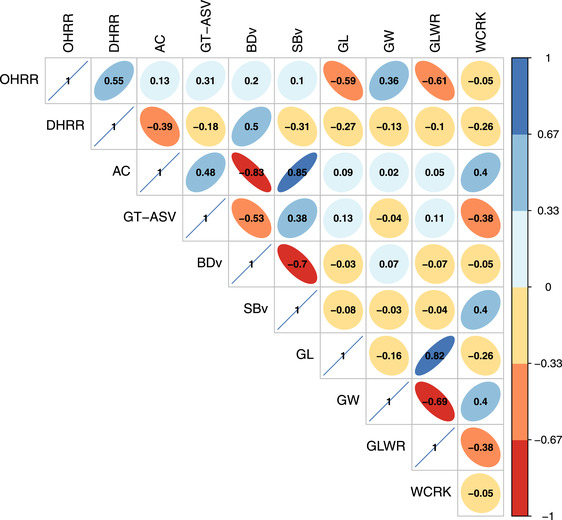
Genetic correlations between grain quality traits. Genetic correlation heat map between 10 grain quality traits OHRR (optimal head rice recovery), DHRR (delayed head rice recovery), AC (amylose‐content), GT–ASV (gelatinization‐temperature expressed as alkali‐spread value), BDv (breakdown‐viscosity), SBv (setback‐viscosity), GL (grain length), GW (grain width), GLWR (grain length to width ratio), and WCRK (white core rice kernel) evaluated on 284 VIOFLAR Informative Panel lines across two different seasons

Broad sense heritability estimates (*H* ^2^) for each trait evaluated across two seasons varied from 0.7 to 0.96 with a mean of 0.87 (Supplemental Table S2). Genetic correlations between grain quality traits are summarized in Figure 2, Supplemental Table S2, and Supplemental Figure S2. Genetic correlations between the 10‐grain quality traits ranged from −0.83 to 0.85 (Supplemental Table S2). Based on a Neighbor‐Joining tree estimated using the genetic correlation matrix, five clusters of traits were identified showing similar relationships between them with the first group made up of AC, GT–ASV, and SBv. The second had GL and GLWR, the third had GW and WCRK, the fourth had DHRR and BDv, and the fifth OHRR (Supplemental Figure S2).

### 3.3 Genetic correlation with milling quality traits OHRR and DHRR

The grain milling quality trait OHRR showed positive genetic correlations with DHRR (0.55), GW (0.36), GT–ASV (0.31), BDv 0.2), AC (0.13), and SBv (0.1). The trait OHRR had negative genetic correlation with GL (−0.59). The trait DHRR had positive genetic correlations with OHRR (0.55) and BDv (0.5), and negative genetic correlation with AC (−0.39), GL (−0.27), WCRK (−0.26), GT–ASV (−0.18), and GW (−0.13) (Figure 2 and Supplemental Table S2).

### 3.4 Genetic correlation with cooking quality traits AC, GT–ASV, BDv, and SBv

The grain quality traitAC showed positive genetic correlations with SBv (0.85),GT–ASV (0.48), WCRK (0.4), and OHRR (0.13) and negative genetic correlations with BDv (−0.83) and DHRR (−0.39). The grain quality trait GT–ASV showed positive genetic correlations with AC (0.48), SDv (0.38), OHRR (0.31), and GL (0.13) and negative genetic correlations with BDv (−0.53), WCRK (−0.38), and DHRR (−0.18) (Figure 2, Supplemental Table S2). The cooking quality trait SBv had positive genetic correlations with AC (0.85), WCRK (0.4), GT–ASV (0.38), and OHRR (0.1) and negative genetic correlations with BDv (−0.7) and DHRR (−0.31). Breakdown viscosity showed positive genetic correlations with DHRR (0.5) and OHRR (0.2) and negative genetic correlations with AC (−0.83), SBv (−0.7), and GT–ASV (−0.53) (Figure 2 and Supplemental Table S2).

### 3.5 Genetic correlation with appearance traits GL, GW, GLWR, and WCRK

The grain appearance trait GL showed a negative genetic correlation with OHHR (−0.59), DHRR (−0.27), and GW (−0.16). Positive genetic correlations were observed between GW with WCRK (0.48) and OHRR (0.36). Negative genetic correlations were observed between GW and DHRR (−0.13). Negative genetic correlations were observed between OHRR and GLWR (−0.61) and positive genetic correlations were observed between GLWR and GT–ASV (0.11). The appearance trait WCRK showed positive genetic correlations with SBv (0.4), AC (0.4), and GW (0.48), and negative genetic correlations with GT–ASV (−0.38) and DHRR (−0.26) (Figure 2 and Supplemental Table S2).

### 3.6 VIP cluster analysis based on OHRR, AC, GT–ASV, BDv, SBv, GLWR, and WCRK phenotypes

**FIGURE 3:**
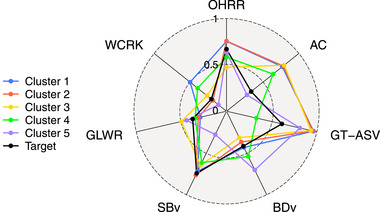
Trait mean values for each phenotypic cluster group. Boxplots for (A) OHRR (optimal head rice recovery), (B) AC (amylose‐content), (C) gelatinization‐temperature (GT), (D) BDv (breakdown‐viscosity), (E) SBv (setback‐viscosity), (F) GLWR (grain length to width ratio), and (G) WCRK (white core rice kernel) for each cluster identified using gap statistic. The target values for each trait are OHRR ≥60% (or ≥0.66), AC ≥25.5% (or ≥0.33), GT–ASV (gelatinization‐temperature expressed as alkali‐spread value) ≥5 (or 0.6), BDv ≤800 (or ≤0.41), SBv ≥1,600 (or 0.74), GLWR ≥3.2 mm (or 0.36), and WCRK ≤7.08% (or ±0.2)

The VIP lines were clustered using seven quality traits: OHRR,AC GT–ASV,BDv, SBv, GLWR, and WCRK. These traits represent typical targets for breeding and rice marketing. Using a Gap Statistic test, five different groups or clusters were identified (Supplemental Figure S3A and Supplemental Table S3). Mean BLUE values for each group for the traits OHRR, AC, GT–ASV, BDv,SBv, GLWR, and WCRK are summarized in Figure 3, and Supplemental Figure S3B. Each cluster's mean values were compared with the target quality values defined for each trait by FLAR. The VIP lines in Cluster 2 contained most of the lines that fall within FLAR's quality breeding targets (Figure 3). Clusters 1 and 3 meet most of the desired quality profiles with the exception of low WCRK and OHRR values, respectively (Figure 3). Clusters 4 and 5 on average did not meet most of the grain quality requirements defined by FLAR (Figure 3).

A PCA analysis using OHRR, AC, GT–ASV, BDv,SBv, GLWR, and WCRK phenotypes showed that the first PC explained 36% of the total phenotypic variance. The first PC separated lines in Group 5 from the rest (Supplemental Figure S4A). The second PC, explaining 20% of the total phenotypic variation, split Groups 1 and 3 (Supplemental Figure S4B). The third PC, explaining 18% of the total phenotypic variance, separated Group 4 from the others (Supplemental Figure S4C).

### 3.7 Grain quality GWAS

**FIGURE 4:**
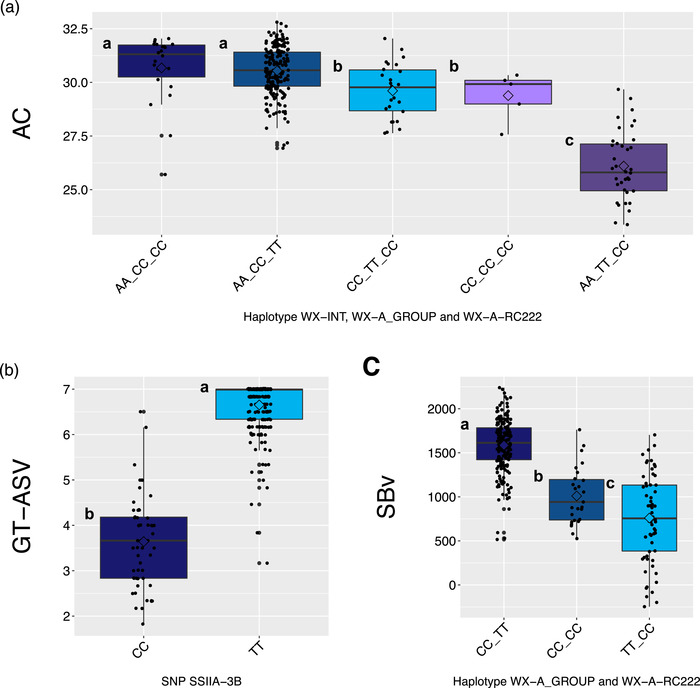
Haplotype phenotypic effect for markers significantly associated with AC (amylose‐content), GT–ASV (gelatinization‐temperature expressed as alkali‐spread value), and BDv (breakdown‐viscosity). Best linear unbiased estimator boxplot regressed on genotypic haplotypes defined by GWAS significant single nucleotide polymorphisms identified for (A) AC, (B) GT–ASV, and (C) SBv (setback‐viscosity). Different letters on each boxplot represent significant differences (*P* < .05) from a multiple HSD Tukey comparison test

The GWAS on OHRR, DHRR, AC, GT–ASV, BDv,SBv, GL, GW, GLWR, and WCRK evaluated in the VIP lines identified 19 marker/trait combinations significantly associated with OHRR, DHRR, AC, GT–ASV, BDv,SBv, GLWR, and WCRK (Table 1, Supplemental Figure S5A–H). Two peaks on chromosome 5 and 6 tagged by the markers chr05:21494622 and WX‐A‐GROUP were associated withDHRR and one marker in chromosome 9, chr09:12326525, was associated with OHRR (Table 1, Supplemental Figure S5A–B). Three markers in chromosome 6 were determined to be significantly associated with AC (Table 1, Supplemental Figure S5C). These three markers defined two distinct peaks, one with the markers WX‐A_GROUP and WX‐A‐RC222 and the other peak tagged by the marker chr06:4641044. Haplotypes groups for AC were defined using markers WX‐A_GROUP, WX‐A‐RC222, and WX‐INT. Even though WX‐INT was not significantly associated with AC in this GWAS, it was included in the analysis because these three markers were described by Dobo et al. (2010) to determine different waxy allelic forms. When haplotypes of these three markers were regressed against AC phenotypic values, the proportion of phenotypic value explained was 0.6 (Figure 4A, Supplemental Table S5). The marker SSIIA‐3B was significantly associated with GT–ASV explaining 0.75 of the phenotypic variation (Figure 4B, Supplemental Figure S5D, and Supplemental Table S5). Two significant markers defining two distinct peaks for BDv were determined by the markers on chromosome 6, WX‐A‐RC222, and SSIIA‐3B, respectively (Table 1, Supplemental Figure S5E). The trait SBv had two significant peaks, one in chromosome 6 between the markers WX‐A_GROUP and WX‐A‐RC222, and one in chromosome 2 defined by the marker chr02:5836334 (Table 1, Supplemental Figure S5F). When haplotypes of two SBv significant markers in chromosome 6 were regressed against SBv phenotypic values, the proportion of phenotypic value observed was 0.51 (Figure 4C, Supplemental Table S5). The traits GLWR and WCRK, each had one peak defined by the markers chr07:25339609 and SSIIA‐3B, respectively (Table 1, Supplemental Figure S5G–H).

**TABLE 1:** Grain quality genome‐wide association studies

SNP marker	Chr	Position	Marker additive effect	−log_10_(*P* value)	PEV	Trait
chr09:12326525	9	12326525	0.35923339	4.40587504	0.03	OHRR
chr05:21494622	5	21494622	0.38106586	5.77064524	0.05	DHRR
WX‐A_GROUP	6	1768724	0.35100042	5.43557854	0.06	DHRR
WX‐A_GROUP	6	1768724	−0.6184418	18.2071211	0.41	AC
WX‐A‐RC222	6	1768998	0.41981083	8.77593029	0.27	AC
chr06:4641044	6	4641044	0.35791404	5.02910397	0.17	AC
SSIIA‐3B	6	6752888	0.94434899	47.0745304	0.76	GT–ASV
WX‐A_GROUP	6	1768724	0.48251521	11.5536241	0.17	BDv
SSIIA‐3B	6	6752888	−0.2894151	5.1736761	0.07	BDv
WX‐A_GROUP	6	1768724	−0.4985356	11.9736197	0.4	SBv
WX‐A‐RC222	6	1768998	0.59671931	17.0275116	0.49	SBv
chr07:25339609	7	25339609	0.28560728	4.47817851	0.19	GLWR
SSIIA‐3B	6	6752888	−0.2182546	4.32079601	0.06	WCRK

*Note*. Marker significantly associated, defined using a Bonferroni multiple testing correction *P* < .05, for eight different grain quality traits evaluated in the VIOFLAR Informative Panel (VIP) set. AC, amylose‐content; BDv, breakdown‐viscosity; Chr, chromosome; DHRR, delayed head rice recovery; GLWR, grain length to width ratio; GT–ASV, gelatinization‐temperature expressed as alkali‐spread value; OHRR, optimal head rice recovery; PEV, phenotypic explain variation; SBv, setback‐viscosity; SNP, single nucleotide polymorphism; WCRK, white core rice kernel.

### 3.8 Grain quality GS

**FIGURE 5:**
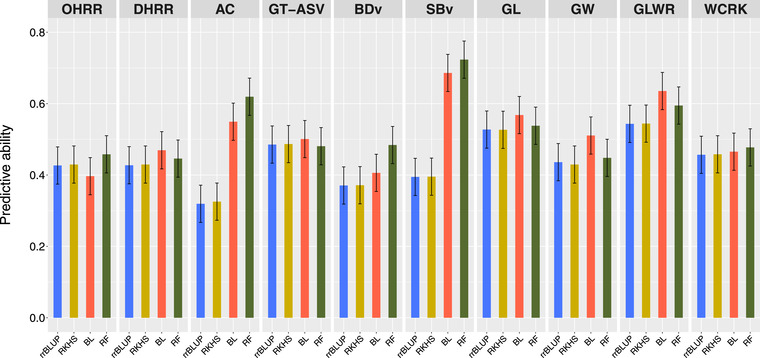
Grain quality genomic selection cross‐validation analysis. Genomic selection fivefold cross validation analysis for 10 grain quality traits evaluated in VIP (VIOFLAR Informative Panel) lines from the 1999 to 2015 yr‐nurseries using four genomic selection models: ridge regression (*rrBLUP*), Reproducible Kernel Hilbert Space (RKHS), Bayesian LASSO (BL) and random forest (RF). GT–ASV, gelatinization‐temperature expressed as alkali‐spread value; SBv, setback‐viscosity; SNP, single nucleotide polymorphism

Genomic selection cross‐validation analyses were performed using four different models: rrBLUP, RKHS, BL, and RF in 10 different grain quality traits using the VIP lines. The results were summarized in Figure 5 and Supplemental Table S4. The predictive abilities of each trait ranged from 0.4 to 0.45 for OHRR, 0.42 to 0.47 for DHRR, 0.3 to 0.6 for AC, 0.48 to 0.5 for GT–ASV, 0.37 to 0.5 for BDv, 0.39 to 0.72 for SBv, 0.52 to 0.56 for GL, 0.42 to 0.51 for GW, 0.54 to 0.63 for GLWR, and 0.45 to 0.47 for WCRK (Figure 5 and Supplemental Table S4). In general, RF had the highest predictive abilities across all traits (Figure 5 and Supplemental Table S4) and had significant better (*P* value <.05) predictive abilities for AC, BDv, and SBv compared with rrBLUP and RKHS (Supplemental Table S4). The model RF outperformed rrBLUP predictive ability by 30, 11, and 32% for AC, BDv, and SBv, respectively (Figure 5, Supplemental Table S4).

### 3.9 Total and additive genetic trends for grain quality traits

**FIGURE 6:**
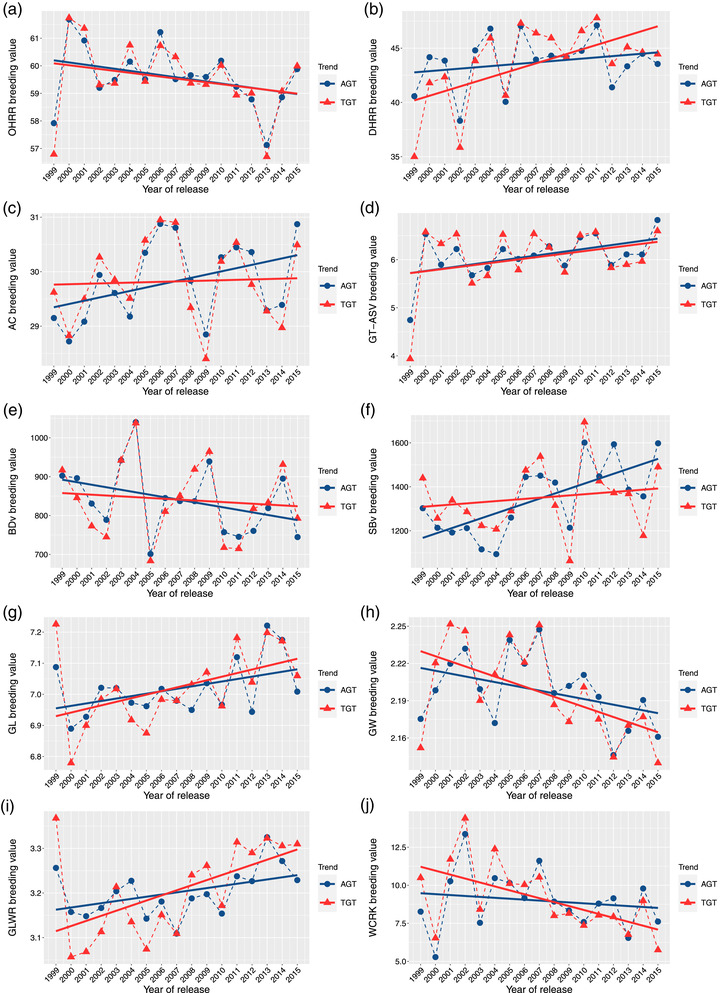
Genetic trends for traits OHRR (optimal head rice recovery), DHRR (delayed head rice recovery), AC (amylose‐content), GT–ASV (gelatinization‐temperature expressed as alkali‐spread value), BDv (breakdown‐viscosity), SBv (setback‐viscosity), GL (grain length), GW (grain width), GLWR (grain length to width ratio), and WCRK (white core rice kernel) across 20 years. Additive (AGT, in blue) and total (TGT, in red) genetic gain for (A) OHRR, (B) DHHR, (C) AC, (D) GT–ASV, (E) BDv, (F) SBv, (G) GL, (H) GW, (I) GLWR, and (J) WCRK

Positive significant total genetic trends (*P* value <.05) were observed for DHRR, GL, and GLWR with an annual increment of 1.0, 0.2, and 0.45%, respectively (Figure 6B, G, and I, Supplemental Table S6). Negative significant total genetic trends (*P* value <.05) were observed for OHRR, GW, and WCRK with an annual percentage decrease of 0.16, 0.21, and 2.4%, respectively (Figure 6A, H, and J, Supplemental Table S6).

Positive significant additive genetic trends (*P* value <.05) were observed for SBv with an annual increment of 2.15% (Figure 6F, Supplemental Table S6). Negative significant additive genetic trends (*P* value <.05) were observed for OHRR, BDv, and GW with an annual percentage decrease of 0.16, 1.0, and 0.16%, respectively (Figure 6A, E, and H, Supplemental Table S6).

Positive but not significant total and additive genetic trends (*P* value <.05) were observed for AC and GT–ASV (Figure 6C and D, Supplemental Table S6).

## 4 DISCUSSION

The PCA of genotypic data in this study showed no major subpopulation structure among the VIP lines. These results were similar to previous studies performed in *indica* rice breeding programs. Begum et al. (2015) evaluated *indica* breeding lines from the International Rice Research Institute's Irrigated Rice Program and found that the first three PC explained 10% of the total genetic variance when 73,147 SNPs were used to determine the relationship between lines. The population structure results indicated that VIP lines belonged to a single population of *indica* lines, consistent with FLAR's intrapopulation improvement breeding strategy where the introduction of external germplasm has been constrained. Subtler degree differences among VIP lines were observed when a Neighbor‐Joining tree constructed using a pairwise *F*_ST_ matrix among VIOFLAR nurseries was generated. Three groups consisting of VIP lines from 1999, 2000 to 2005, and 2003 and 2006to 2015 were observed. Based on FLAR's breeding history described by Berrio‐Orozco et al., 2016, the VIP lines from 1999 were derived from the first crosses FLAR did after its creation in 1995. The base population consisted of lines collected from different programs in South America and Asia. The second group from 2000 to 2005 was derived mostly from crosses between CIAT lines aiming to improve adaptability and disease resistance for tropical regions in South America. The third group made up of VIP nurseries from 2006 to 2015 were derived from FLAR–FLAR, and FLAR–CIAT crosses. Ideotype breeding objectives were considered during the development and selection of the third group nurseries. Interestingly, most of the crosses that generated the nurseries from 2006 to 2015, were derived from the 2003 VIOFLAR that clustered with this group (Berrio‐Orozco et al., 2016).

A PCA using the phenotypic values of OHRR, AC, GT–ASV, BDv,SBv, GLWR, and WCRK was also conducted in this study. In contrast to the genotypic PCA results, the two first PCs explained 56% of the total variance. This was significantly higher than the proportion explained by the genotypic data. The larger proportion of variation explained by the phenotypes compared with the genotypic data can be explained by the presence of major effect QTLs for different traits. This was confirmed by the GWAS analysis, especially for the traits AC, GT–ASV, BDv, and SBv where large effect SNPs were associated with these traits. A multivariate cluster analysis using the same grain quality phenotypes grouped the VIP lines in five clusters. Cluster 2, which accounts for 30% of the VIP lines, met, on average, all the minimum grain quality standards defined by FLAR for different LAC markets. These quality standards were defined as OHRR ≥60%, AC ≥26.5%, GT–ASV ≥5, BDv ≤800, SBv ≥1,600, GLWR ≥3.2, and WCRK ≤7.08%. The VIP lines in Cluster 2 are a source of germplasm to develop cultivars for most LAC rice markets.

Improvement of genotypic value for different grain quality traits is essential to develop better cultivars that meet the quality demands of LAC rice markets. To better understand the total and additive genetic progress of FLAR's breeding program for different grain quality traits we estimated genetic trends for the past 20 yr. Positive and negative significant (*P* value <.05) desired genetic trends were observed for DHHR, BDv, SBv, GL, GW, and GLWR. These results showed the effectiveness of FLAR's breeding program to breed germplasm with the desire grain appearance, milling, and cooking qualities. No significant effects in the genetic trend were observed for AC and GT–ASV. This can be explained by early selection and maintenance of germplasm with high AC and GT–ASV values. These traits were prioritized early in the breeding program. Evidence of this was observed in the phenotypic cluster analysis where every cluster except number 5 and 4 met the minimum requirements for AC and GT–ASV, respectively. Negative undesired significant genetic trends were observed for OHRR. Increasing values’ chalkiness, WCRK, and GL have been associated with increased breakage during milling (Counce et al., 2005). A negative genetic correlation with GLWR may partially explained this trend where the selection of grains with higher GLWR values may unintentionally decrease overall levels of OHRR. In addition, high genotype × environment effect observed for OHRR can decrease the progress of selection. Routine evaluation of OHRR in combination with novel strategies such as MAS and GS that includes genotype × environment interactions can improve the rate of genetic gain for OHRR.

Our GWAS in the VIP lines identified three SNPs significantly associated with AC. Two of these SNPs, WX‐A_GROUP, and WX‐A‐RC222, located on chromosome 6, are diagnostic markers that detect different functional alleles in exon 6 and 10 of the *Waxy* gene (*Wx*) (Dobo et al., 2010; Teng et al., 2017). *Waxy* encodes for the GBSS I enzyme that is responsible for amylose synthesis (Hanashiro et al., 2008). The third SNP, chr06:4641044, colocalized with two QTLs (AAC_MTA3 and AAC_MTA4) reported in a previous GWAS for different starch parameters in rice (Biselli et al., 2019). The authors concluded that QTLs AAC_MTA3 and AAC_MTA4 were in the same LD block as *Waxy* (Biselli et al., 2019). Our findings showed that gene *Waxy* was associated with the GWAS peak, explaining a large proportion of the phenotypic variation for AC. Haplotypes constructed using the three functional *Waxy* markers; WX‐INT, WX‐A_GROUP, and WX‐A‐RC222 explained 60% of the phenotypic variances of AC in the VIP lines. These three markers, WX‐INT, WX‐A_GROUP, and WX‐A‐RC222, can be used for MAS strategies to select germplasm with intermediate and high amylose in FLAR's breeding program.

The SNPs WX‐A_GROUP and WX‐A‐RC222 were also found significantly associated with DHRR, BDv, and SBv. Different studies have demonstrated that the molecular properties of AC, such as chain length, branching ratio, and molecular mass, affect rice pasting viscosity, rate of starch retrogradation, gel texture, and chalkiness (Gidley & Bulpin, 1989; Mua & Jackson, 1997). Xu et al. (2016) conducted a GWAS of eating and cooking qualities in *indica* rice and identified makers linked to *Waxy* associated with BDv and SDv. Our study validated Xu et al.’s (2016) GWAS results. Functional *Waxy* allele markers explained 17 and 49% of the phenotypic variation for BDv and SDv, respectively. The cooking quality trait SBv is related to starch retrogradation tendencies after gelatinization and cooling periods (Shafie et al., 2016). Using haplotypes defined with the two markers identified in this GWAS, WX‐A_GROUP, and WX‐A‐RC222, lines can be grouped into high, intermediate, and low SBv in MAS strategies. The *Waxy* gene not only play a key role in controlling eating and cooking quality but also influences other traits including HRR. Zhou et al. (2015) showed that the *Waxy* region was significantly associated with HRR. Our results showed that *Waxy* was particularly important when HRR was measured under delayed harvest conditions (DHRR). The pleiotropic effects of *Waxy* on AC, BDv, SBv, and DHRR can explain the higher genetic correlations observed among these traits and the intricate relationships between cooking and milling quality traits in rice.

The diagnostic marker SSIIA‐3B tagging the *SSIIa* gene was significantly associated with GT–ASV, explaining 76% of the total phenotypic variation for GT in the VIP lines. The *SSIIa* gene is involved in the synthesis of intermediate amylopectin chains (Bao et al., 2014). The reduction or complete loss of *SSIIa* can alter the composition and amount of starch in the endosperm (Luo et al., 2015). Our GWAS showed that *SSIIa* not only affect GT–ASV, but it also has significant effects in the phenotypic variation of BDv and WCRK. According to Luo et al. (2015) and Butardo et al. (2020), the phenotypic outcome of *SSIIa* variation include elevation in AC, increase in the amount of short amylopectin chains resulting in the proportional decrease in the intermediate chains, reduction of amylopectin content, lowering gelatinization‐temperature, and alterations in starch crystallinity and viscosity, which is consistent with the observed association between BDv and the functional *SSIIa* marker SSIIA‐3B. The gene *SSIIa* has been reported to be a major factor explaining up to 17% of the degree of endosperm chalkiness in a panel of 375 advance *indica* lines (Zhao et al., 2015). Our results confirmed previous findings by showing the significant association between the gene *SSIIa* and WCRK, which is a type of chalkiness. The large phenotypic effect explained by SNP SSIIA‐3B on GT–ASV makes it an ideal marker for MAS strategies to differentiated FLAR's germplasm with low and high‐intermediate to high GT. Again, the pleiotropic effects of *SSIIa* on GT–ASV, BDv, and WCRK can partially explained the high genetic correlation observed among these three traits.

Three novel QTLs were found on chromosomes 9, 5, and 7 associated with OHHR, DHRR, and GLWR, respectively. According to Bao (2014), up to 34 QTLs located at all chromosomes have been reported in 10 studies for HRR. The new QTLs in chromosome 9 and 5 explained 3 and 5% of the phenotypic variation for OHHR and DHRR. These results confirm the complex genetic architecture of HRR. For the trait GLWR, the locus on chromosome 7, defined by a SNP located at 25,339,609 bp, was found to be significantly associated. This region is ∼1 Mb upstream of *qGL7‐2* (Shao et al., 2010), a region associated with GL in rice. Further genetic mapping studies need to be carried out to determine if these two QTLs are the same or are explained by different genetic factors. The SNP chr07.25339609, explaining 19% of the phenotypic variation for GLWR, is a candidate for MAS to detect lines with high GLWR. The grain shape qualities GL and GW had the lower standard deviations among the quality traits measured in the VIP with average values of 7.01 and 3.2 mm and standard deviations of 0.3 and 0.2 mm, respectively. The 1k‐RiCA contains a trait marker associated with grain size and shape. The marker ‘GS3’ for grain size, located in chromosome 3 (Mao et al., 2010), has the desired allele T for long‐grain fixed in the VIP lines.

As we showed, some of the loci identified in this GWAS can explained the complex genetic correlation among different grain quality traits. The degree of genetic correlation among these traits can enhance or diminish progress from selection. This study found that AC had a positive genetic correlation with WCRK and a negative genetic correlation with DHRR. Besides germplasm with high AC, FLAR aims to develop cultivars with low WCRK (≤0.8) and high DHRR (≥57%) These correlations indicated that, either by pleiotropic or linkage effects, selecting higher AC could select lines with undesired WCRK and DHRR values. This study showed that the diagnostic *Waxy* allelic marker WX‐A_GROUP was associated with AC and DHRR showing evidence of pleiotropy effects explaining the genetic correlations observed. This GWAS identified the marker SSIIA‐3B in chromosome 6 significantly associated with WCRK. The marker SSIIA‐3B is located 19 cM away from WX‐A_GROUP, showing evidence of linkage and possibly partially explaining the genetic correlation between both traits. Despite these undesired correlations, FLAR has been able to significantly improve the total genetic gain for DHRR and WCRK. Careful evaluation of AC, DHRR, and WCRK has allowed FLAR to identify recombinants with desired AC, DHRR, and WCRK. In contrast, desired positive genetic correlation between AC, GT–ASV, OHHR, and SBv were consistent to previous studies (Pang et al., 2016).

Through cross‐validation analysis this study explored the possibility for implementing GS to improve grain milling, quality, and appearance traits in FLAR's breeding program. Prediction accuracies of 0.45, 0.47, 0.6, 0.5, 0.5, 0.72, 0.56, 0.51, 0.63, and 0.47 for OHHR, DHRR, AC, GT–ASV, BDv, SBv, GL, GW, GLWR, and WCRK, respectively, demonstrated the potential to implement GS in FLAR's breeding strategies for grain quality. Overall, higher prediction accuracies were observed when BL and RF were implemented. Grenier et al. (2015) described that higher prediction accuracies by BL were the consequence of fitting a reduced number of explanatory variables to the model, fitting some markers to zero effect, and making it possible to avoid overparameterization for a trait controlled by a few major loci as it was shown by the GWAS results. Nonlinear methods such as RF have been shown to effectively captured large‐effect QTL in rice (Spindel et al., 2015). These results suggest that models such as BL and RF can be used when large effect QTLs are known to be segregating for grain quality traits. In addition, RF has been shown to work best with smaller number of markers (Spindel et al., 2015), ideally when low‐density cost‐effective genotypic platforms are used. This study can be expanded in future studies to implement different models that include genotype × genotype and genotype × environment large effect QTLs and training set optimization to improve prediction abilities.

This study provided a pathway to effectively implement MAS and GS to improve grain milling, appearance, cooking, and edible qualities for rice markets in LAC. The markers identified in this study associated with AC, GT–ASV, and SBv can be deployed at different genotyping service providers to integrate forward breeding strategies and perform selection at early stages of the breeding program. The cost‐effective genotyping platform 1k‐RiCA can be routinely used in GS strategies in FLAR's breeding program. Further studies need to be done to design the best strategy to deploy GS in FLAR's breeding program that complement the current phenotypic selection strategies to develop rice cultivars with specific grain quality profiles suited for LAC and different export markets.

## AUTHOR CONTRIBUTIONS

Maribel Cruz: Conceptualization; Data curation; Formal analysis; Investigation; Methodology; Project administration; Resources; Supervision; Writing‐original draft; Writing‐review & editing. Juan David Arbelaez: Conceptualization; Data curation; Formal analysis; Funding acquisition; Investigation; Methodology; Project administration; Resources; Supervision; Writing‐original draft; Writing‐review & editing. Katherine Loaiza: Methodology; Resources; Writing‐review & editing. Juan Rosas: Conceptualization; Investigation; Methodology; Writing‐review & editing. Juan Cuasquer: Data curation; Formal analysis; Methodology; Resources. Eduardo Graterol: Conceptualization; Funding acquisition; Project administration; Resources; Supervision; Writing‐original draft; Writing‐review & editing.

## CONFLICT OF INTEREST

The authors declare no conflict of interests.

## Supporting information


**Supplemental Figure S1**. VIOFLAR Informative Panel (VIP) principal component analysis. PCA of 284 VIP (VIOFLAR Informative Panel) lines from the 1999 to 2015 year‐nurseries using the genotypic data generated with the 1k‐RiCA SNP assay on each line. **A)** Scatter plot of PC1 vs. PC3, **B)** scatter plot of PC2 vs. PC3
**Supplemental Table S1**. Weir and Cockerham's *F_ST_* table between the VIOFLAR's nurseries from 1999 to 2015.
**Supplemental Table S2**. Broad sense heritably (*H^2^*) and correlation between grain quality traits. Genetic (g.Cor) and phenotypic (p.Cor) correlation table between 10 grain quality traits, OHRR, DHRR, AC, GT−ASV, BDv, SBv, GL, GW, GLWR, and WCRK evaluated on 284 VIP lines across two different seasons.
**Supplemental Figure S2**. Genetic correlation represented using an unrooted Neighbor‐Joining tree between OHRR, DHRR, AC, GT−ASV, BDv, SBv, GL, GW, GLWR, and WCRK.
**Supplemental Figure S3**. **A)** Gap Statistic test using BLUEs phenotypic matrix with the traits OHRR, AC, GT−ASV, BDv, SBv, GLWR, and WCRK. **B)** Mean values and standard deviations for OHRR, AC, GT−ASV, BDv, SBv, GLWR, and WCRK for each cluster identified.
**Supplemental Table S3**. VIP lines grouped in 5 different clusters based on the Gap Statistic method using the phenotypic data of, OHRR, AC, GT−ASV, BDv, SBv, GLWR, and WCRK (‘Group’ from 1 to 5)
**Supplemental Figure S4**. PCA of traits OHRR, AC, GT−ASV, BDv, SBv, GLWR, and WCRK evaluated in the VIP color coded by each group.
**Supplemental Figure S5**. Grain quality Genome Wide Association Studies (GWAS). GWA Manhattan and Q‐Q plots for **A)** optimal‐head‐rice‐recovery (OHRR), **B)** delayed‐head‐rice‐recovery (DHRR), **C)** amylose‐content (AC) **D)** gelatinization‐temperature expressed as alkali‐spread value (GT−ASV), **E)** breakdown‐viscosity (BDv) **F)** setback‐viscosity (SBv), **G)** grain‐length‐to‐width‐ratio (GLWR) and **H)** white‐core‐rice‐kernel (WCRK).
**Supplemental Table S4**. Genomic selection cross validations analysis for 10 grain quality traits using four GS models, ridge regression (*rrBLUP*), reproducing kernel Hilbert space (*RKHS*), Bayesian LASSO (*BL*) and random forest (*RF*)
**Supplemental Table S5**. Haplotype analysis for AC, GT−ASV, and SBv.
**Supplemental Table S6**. Grain quality Total and Additive genetic trend table.

Supplementary Material
