# Supplementary material for: Genetic and phenotypic characterization of rice grain quality traits to define research strategies for improving rice milling, appearance, and cooking qualities in Latin America and the Caribbean
Source: Plant Genome. 2021 Sep 12;14(3):e20134. doi: 10.1002/tpg2.20134 (PMC12807091; doi:10.1002/tpg2.20134)
Supplement: Supplementary file 1 [file TPG2-14-e20134-s002.docx]

**Genetic and phenotypic characterization of rice grain quality traits to define research strategies for improving rice cooking qualities in Latin America and The Caribbean**

**Maribel Cruz.** Co-First Author. FLAR (Fondo Latinoamericano para Arroz de Riego), CIAT (International Center for Tropical Agriculture) - Kilómetro 17 Recta Cali – Palmira, CP 763537, Cali, Valle del Cauca – Colombia. Email: maribel.cruz@cgiar.org

**Juan David Arbelaez**. Co-First Author. Corresponding Author. Crop Sciences, University of Illinois, Urbana-Champaign, Turner Hall N-211|1102 S. Goodwin Ave. | 046 Urbana, IL 61801, USA. Email: arbelaez@illinois.edu

**Katherine Loaiza**. FLAR (Fondo Latinoamericano para Arroz de Riego), CIAT (International Center for Tropical Agriculture)- Kilómetro 17 Recta Cali – Palmira, CP 763537, Cali, Valle del Cauca – Colombia. Email: j.k.loaiza@cgiar.org

**Juan Cuasquer**. CIAT (International Center for Tropical Agriculture)- Kilómetro 17 Recta Cali – Palmira, CP 763537, Cali, Valle del Cauca – Colombia. Email: j.b.cuasquer@cgiar.org

**Juan Rosas**. INIA (Instituto Nacional de Investigación Agropecuaria), Ruta 8 Km. 281/33000, Treinta y Tres, Uruguay. Email: jrosas@tyt.inia.org.uy

**Eduardo Graterol**. FLAR (Fondo Latinoamericano para Arroz de Riego), CIAT (International Center for Tropical Agriculture)- Kilómetro 17 Recta Cali – Palmira, CP 763537, Cali, Valle del Cauca – Colombia. Email: e.j.graterol@cgiar.org

**Supplementary Figures and Tables**

**A**

**B**

**Supplemental Figure 1**. VIOFLAR Informative Panel (VIP) principal component analysis. PCA of 284 VIP (VIOFLAR Informative Panel) lines from the 1999 to 2015 year-nurseries using the genotypic data generated with the 1k-RiCA SNP assay on each line. **A)** Scatter plot of PC1 vs. PC3, **B)** scatter plot of PC2 vs. PC3.

**Supplemental Table 1**. Weir and Cockerham’s *F_ST_* table between the VIOFLAR’s nurseries from 1999 to 2015.

|  | 1999 | 2000 | 2001 | 2002 | 2003 | 2004 | 2005 | 2006 | 2007 | 2008 | 2009 | 2010 | 2011 | 2012 | 2013 | 2014 | 2015 | T |
| --- | --- | --- | --- | --- | --- | --- | --- | --- | --- | --- | --- | --- | --- | --- | --- | --- | --- | --- |
| 1999 |  | 0.17 | 0.20 | 0.17 | 0.17 | 0.21 | 0.18 | 0.18 | 0.26 | 0.20 | 0.15 | 0.21 | 0.18 | 0.23 | 0.21 | 0.20 | 0.19 | 0.13 |
| 2000 |  |  | 0.03 | 0.04 | 0.07 | 0.06 | 0.07 | 0.06 | 0.12 | 0.09 | 0.05 | 0.07 | 0.09 | 0.09 | 0.07 | 0.08 | 0.10 | 0.03 |
| 2001 |  |  |  | 0.06 | 0.12 | 0.08 | 0.09 | 0.11 | 0.13 | 0.11 | 0.08 | 0.11 | 0.11 | 0.10 | 0.11 | 0.11 | 0.12 | 0.04 |
| 2002 |  |  |  |  | 0.10 | 0.04 | 0.02 | 0.09 | 0.11 | 0.08 | 0.06 | 0.07 | 0.09 | 0.09 | 0.08 | 0.08 | 0.11 | 0.03 |
| 2003 |  |  |  |  |  | 0.12 | 0.08 | 0.04 | 0.08 | 0.04 | 0.03 | 0.07 | 0.08 | 0.06 | 0.07 | 0.08 | 0.08 | 0.06 |
| 2004 |  |  |  |  |  |  | 0.04 | 0.10 | 0.13 | 0.10 | 0.08 | 0.10 | 0.10 | 0.12 | 0.10 | 0.11 | 0.12 | 0.05 |
| 2005 |  |  |  |  |  |  |  | 0.07 | 0.10 | 0.07 | 0.06 | 0.07 | 0.09 | 0.08 | 0.07 | 0.08 | 0.10 | 0.05 |
| 2006 |  |  |  |  |  |  |  |  | 0.08 | 0.05 | 0.04 | 0.03 | 0.05 | 0.06 | 0.04 | 0.08 | 0.07 | 0.04 |
| 2007 |  |  |  |  |  |  |  |  |  | 0.04 | 0.07 | 0.06 | 0.07 | 0.05 | 0.07 | 0.06 | 0.07 | 0.07 |
| 2008 |  |  |  |  |  |  |  |  |  |  | 0.03 | 0.06 | 0.05 | 0.02 | 0.06 | 0.07 | 0.05 | 0.04 |
| 2009 |  |  |  |  |  |  |  |  |  |  |  | 0.05 | 0.04 | 0.04 | 0.04 | 0.02 | 0.03 | 0.02 |
| 2010 |  |  |  |  |  |  |  |  |  |  |  |  | 0.04 | 0.06 | 0.02 | 0.07 | 0.07 | 0.05 |
| 2011 |  |  |  |  |  |  |  |  |  |  |  |  |  | 0.03 | 0.04 | 0.05 | 0.04 | 0.04 |
| 2012 |  |  |  |  |  |  |  |  |  |  |  |  |  |  | 0.04 | 0.05 | 0.04 | 0.05 |
| 2013 |  |  |  |  |  |  |  |  |  |  |  |  |  |  |  | 0.06 | 0.07 | 0.05 |
| 2014 |  |  |  |  |  |  |  |  |  |  |  |  |  |  |  |  | 0.03 | 0.04 |
| 2015 |  |  |  |  |  |  |  |  |  |  |  |  |  |  |  |  |  | 0.03 |
| T |  |  |  |  |  |  |  |  |  |  |  |  |  |  |  |  |  |  |

**Supplemental Table 2**. Broad sense heritably (*H^2^*) and correlation between grain quality traits. Genetic (g.Cor) and phenotypic (p.Cor) correlation table between 10 grain quality traits, $OHRR$, $DHRR$, $AC$, $GT-ASV$, $BDv$, $SBv$, $GL$, $GW$, $GLWR$, and $WCRK$ evaluated on 284 VIP lines across two different seasons. Significant level with P-values les or equal 0.05 and 0.01 were defined by * and ** respectively.

| Trait | *H^2^* | Correlation | *OHRR* | *DHRR* | *AC* | *GT-ASV* | *BDv* | *SBv* | *GL* | *GW* | *GLWR* | *WCRK* |
| --- | --- | --- | --- | --- | --- | --- | --- | --- | --- | --- | --- | --- |
| *OHRR* | **0.78** | **p.Cor** | 1.00 | 0.35 | 0.06 | 0.18 | 0.01 | 0.14 | -0.37 | 0.29 | -0.42 | -0.04 |
|  |  | **g.Cor** | 1.00 | 0.55 | 0.13 | 0.31 | 0.20 | 0.10 | -0.59 | 0.36 | -0.61 | -0.05 |
| *DHRR* | **0.8** | **p.Cor** | 0.35** | 1.00 | -0.22 | -0.03 | 0.21 | -0.11 | -0.15 | -0.05 | -0.07 | -0.28 |
|  |  | **g.Cor** | 0.55** | 1.00 | -0.39 | -0.18 | 0.50 | -0.31 | -0.27 | -0.13 | -0.10 | -0.26 |
| *AC* | **0.89** | **p.Cor** | 0.06 | -0.22* | 1.00 | 0.30 | -0.64 | 0.75 | -0.02 | 0.10 | -0.07 | 0.23 |
|  |  | **g.Cor** | 0.13 | -0.39** | 1.00 | 0.48 | -0.83 | 0.85 | 0.09 | 0.02 | 0.05 | 0.40 |
| *GT-ASV* | **0.82** | **p.Cor** | 0.18* | -0.03 | 0.30** | 1.00 | -0.38 | 0.21 | 0.02 | 0.04 | -0.01 | -0.21 |
|  |  | **g.Cor** | 0.31** | -0.18* | 0.48** | 1.00 | -0.53 | 0.38 | 0.13 | -0.04 | 0.11 | -0.38 |
| *BDv* | **0.91** | **p.Cor** | 0.01 | 0.21* | -0.64** | -0.38** | 1.00 | -0.53 | 0.12 | -0.01 | 0.09 | 0.02 |
|  |  | **g.Cor** | 0.20* | 0.50** | -0.83** | -0.53** | 1.00 | -0.70 | -0.03 | 0.07 | -0.07 | -0.03 |
| *SBv* | **0.95** | **p.Cor** | 0.14 | -0.11 | 0.75** | 0.21* | -0.53** | 1.00 | -0.11 | 0.08 | -0.12 | 0.11 |
|  |  | **g.Cor** | 0.10 | -0.31** | 0.85** | 0.38** | -0.70** | 1.00 | -0.08 | -0.03 | -0.04 | 0.07 |
| *GL* | **0.95** | **p.Cor** | -0.37** | -0.15 | -0.02 | 0.02 | 0.12 | -0.11 | 1.00 | -0.32 | 0.84 | 0.00 |
|  |  | **g.Cor** | -0.59** | -0.27** | 0.09 | 0.13 | -0.03 | -0.08 | 1.00 | -0.16 | 0.82 | 0.09 |
| *GW* | **0.96** | **p.Cor** | 0.29** | -0.05 | 0.10 | 0.04 | -0.01 | 0.08 | -0.32** | 1.00 | -0.79 | 0.34 |
|  |  | **g.Cor** | 0.36** | -0.13 | 0.02 | -0.04 | 0.07 | -0.03 | -0.16 | 1.00 | -0.69 | 0.48 |
| *GLWR* | **0.7** | **p.Cor** | -0.42** | -0.07 | -0.07 | -0.01 | 0.09 | -0.12 | 0.84** | -0.79** | 1.00 | -0.20 |
|  |  | **g.Cor** | -0.61** | -0.10 | 0.05 | 0.11 | -0.07 | -0.04 | 0.82** | -0.69** | 1.00 | -0.22 |
| *WCRK* | **0.87** | **p.Cor** | -0.04 | -0.28** | 0.23* | -0.21* | 0.02 | 0.11 | 0.00 | 0.34** | -0.20* | 1.00 |
|  |  | **g.Cor** | -0.05 | -0.26** | 0.40** | -0.38** | -0.03 | 0.07 | 0.09 | 0.48** | -0.22* | 1.00 |

**Supplemental Figure 2.** Genetic correlation represented using an unrooted Neighbor-Joining tree between $OHRR$, $DHRR$, $AC$, $GT-ASV$, $BDv$, $SBv$, $GL$, $GW$, $GLWR$, and $WCRK$.

**A**

**B**

| Group | *N* | *OHRR* | *AC* | *GT-ASV* | *BDv* | *SBv* | *GLWR* | *WCRK* |
| --- | --- | --- | --- | --- | --- | --- | --- | --- |
| 1 | 47 | 61.41±1.99 | 30.57±1.12 | 6.85±0.23 | 825.39±139.17 | 1537.82±243 | 3.08±0.18 | 15.13±4.13 |
| 2 | 89 | 61.45±1.67 | 30.69±1.14 | 6.8±0.33 | 722.83±156.56 | 1653.42±286.74 | 3.11±0.14 | 6.51±2.08 |
| 3 | 62 | 58.6±3.29 | 29.36±1.06 | 3.52±0.89 | 995.27±180.61 | 1280.83±352.97 | 3.16±0.2 | 12.29±6.01 |
| 4 | 50 | 56.79±2.35 | 30.82±0.97 | 6.7±0.55 | 640.81±180.63 | 1364.27±392.43 | 3.38±0.2 | 8.76±3.75 |
| 5 | 36 | 59.44±3.68 | 25.94±1.39 | 6.06±0.4 | 1249.87±197.04 | 435.02±342.06 | 3.29±0.27 | 4.4±1.82 |

**Supplemental Figure 3**. **A)** Gap Statistic test using BLUEs phenotypic matrix with the traits $OHRR$, $AC$, $GT-ASV$, $BDv$, $SBv$, $GLWR$, and $WCRK$. **B)** Mean values and standard deviations for $OHRR$, $AC$, $GT-ASV$, $BDv$, $SBv$, $GLWR$, and $WCRK$ for each cluster identified.

**Supplemental Table 3.** VIP lines grouped in 5 different clusters based on the Gap Statistic method using the , $OHRR$, $AC$, $GT-ASV$, $BDv$, $SBv$, $GLWR$, and $WCRK$ (‘Group’ from 1 to 5).

| VIP | OHRR | AC | GT-ASV | BV | SV | GLWR | WCRK | Group | VIOFLAR |
| --- | --- | --- | --- | --- | --- | --- | --- | --- | --- |
| FL00440-47P-2-3P-M | 60.50 | 31.10 | 7.01 | 836.80 | 1894.81 | 3.10 | 12.61 | 1 | 2000 |
| FL00478-29P-5-1P-M | 62.38 | 30.30 | 7.00 | 654.32 | 1577.22 | 3.08 | 12.77 | 1 | 2000 |
| FL00807-17P-3-2P-M | 63.60 | 29.95 | 6.84 | 647.14 | 1636.85 | 2.90 | 18.73 | 1 | 2001 |
| FL00807-9P-5-1P-M | 57.43 | 31.24 | 6.99 | 680.29 | 1707.53 | 3.14 | 19.10 | 1 | 2001 |
| FL00826-5P-4-2P-M | 61.38 | 29.97 | 6.84 | 821.12 | 1130.28 | 3.06 | 15.74 | 1 | 2001 |
| FL00837-4P-5-1P-M | 62.85 | 30.16 | 6.99 | 706.34 | 1333.01 | 2.99 | 13.16 | 1 | 2001 |
| FL00837-8P-5-2P-M | 60.25 | 29.41 | 6.49 | 699.59 | 1629.55 | 2.93 | 17.98 | 1 | 2001 |
| FL01071-18P-1-1P-M | 60.91 | 32.06 | 7.02 | 672.81 | 1541.94 | 2.99 | 11.54 | 1 | 2001 |
| FL01865-1P-9-4P-M | 63.00 | 31.24 | 6.99 | 643.66 | 1726.12 | 3.07 | 11.83 | 1 | 2001 |
| FL01871-19P-3-3P-M | 62.67 | 28.12 | 6.82 | 600.39 | 1334.61 | 2.94 | 17.71 | 1 | 2001 |
| FL02058-24P-2-1P-3-M | 59.34 | 30.99 | 7.00 | 699.11 | 1579.70 | 3.19 | 13.80 | 1 | 2002 |
| FL02087-6P-2-1P-2-M | 61.68 | 30.30 | 6.84 | 748.60 | 1644.58 | 3.15 | 28.78 | 1 | 2002 |
| FL02173-3P-6-5P-2P-M | 61.28 | 30.90 | 6.52 | 781.05 | 1844.26 | 3.06 | 12.50 | 1 | 2002 |
| FL02759-5P-2-1P-1P-M | 59.81 | 30.42 | 7.00 | 854.43 | 1513.95 | 2.88 | 27.83 | 1 | 2002 |
| FL02768-2P-6-1P-1-M | 61.03 | 30.66 | 6.82 | 654.10 | 1551.96 | 3.26 | 18.97 | 1 | 2002 |
| FL02768-2P-6-3P-3-M | 62.24 | 31.92 | 7.01 | 691.18 | 1763.73 | 3.22 | 13.12 | 1 | 2002 |
| FL03191-4P-66-1P-3P-M | 62.93 | 30.93 | 6.99 | 687.97 | 1337.04 | 3.12 | 15.44 | 1 | 2003 |
| FL03194-8P-2-1P-3P-M | 56.87 | 31.91 | 6.98 | 1005.52 | 1397.27 | 3.19 | 14.50 | 1 | 2003 |
| FL03197-15P-10-1P-3P-M | 64.17 | 30.32 | 6.32 | 1075.15 | 1140.82 | 2.91 | 11.36 | 1 | 2003 |
| FL03322-4P-11-1P-2P-M-1P-M | 61.56 | 31.21 | 7.01 | 1013.12 | 1690.49 | 3.30 | 12.49 | 1 | 2004 |
| FL03724-11P-26-4P-2P-M | 63.70 | 30.79 | 7.01 | 905.80 | 1610.03 | 3.15 | 24.66 | 1 | 2004 |
| FL03729-7P-9-1P-1P-M | 61.57 | 27.54 | 6.60 | 964.17 | 709.85 | 3.11 | 14.87 | 1 | 2004 |
| FL03779-8P-14-2P-1P-M | 59.04 | 31.35 | 7.01 | 979.54 | 1656.15 | 3.07 | 14.29 | 1 | 2004 |
| FL04052-2P-3-2P-2P-M | 62.01 | 30.79 | 6.84 | 868.61 | 1563.12 | 3.47 | 13.85 | 1 | 2004 |
| FL04582-5P-1-4P-2P-M | 63.78 | 31.13 | 6.83 | 700.58 | 1367.36 | 2.83 | 15.81 | 1 | 2005 |
| FL04584-5P-12-3P-3P-M | 59.95 | 31.17 | 7.00 | 887.85 | 1590.49 | 3.02 | 17.12 | 1 | 2005 |
| FL04648-6P-4-6P-2P-M | 60.22 | 27.13 | 6.82 | 732.32 | 1170.49 | 2.68 | 11.39 | 1 | 2005 |
| FL04733-5P-2-2P-2P-M | 62.05 | 31.08 | 6.15 | 716.58 | 1760.41 | 3.05 | 17.51 | 1 | 2005 |
| FL04794-6P-4-1P-3P-M | 61.60 | 29.14 | 7.01 | 767.11 | 1149.94 | 3.21 | 12.88 | 1 | 2005 |
| FL05392-3P-12-2P-3P-M | 64.80 | 31.41 | 6.99 | 963.49 | 1408.60 | 3.00 | 16.49 | 1 | 2006 |
| FL05564-8P-1-2P-1P-M | 63.77 | 31.57 | 6.67 | 912.20 | 1723.00 | 3.32 | 13.07 | 1 | 2006 |
| FL06609-9P-4-1P-1P-M | 63.96 | 31.52 | 6.99 | 787.50 | 1354.98 | 2.87 | 11.54 | 1 | 2007 |
| FL06722-1P-2-3P-3P-M | 64.34 | 31.29 | 6.99 | 688.83 | 1495.97 | 3.11 | 12.15 | 1 | 2007 |
| FL06733-16P-1-3P-6P-M | 63.04 | 31.35 | 7.02 | 1034.68 | 1796.62 | 2.89 | 19.21 | 1 | 2007 |
| FL06733-6P-7-2P-2P-M | 63.45 | 31.81 | 7.00 | 878.01 | 1353.39 | 2.91 | 18.61 | 1 | 2007 |
| FL06885-4P-3-2P-1P-M | 58.21 | 29.71 | 6.34 | 781.42 | 1835.59 | 3.17 | 17.93 | 1 | 2007 |
| FL06885-4P-3-2P-4P-M | 58.72 | 29.29 | 7.01 | 961.53 | 1680.92 | 3.55 | 18.31 | 1 | 2007 |
| FL07181-22P-2-2P-1P-M | 59.95 | 30.57 | 6.50 | 919.80 | 1676.01 | 2.92 | 10.38 | 1 | 2008 |
| FL07221-3P-3-3P-1P-M | 60.27 | 31.00 | 7.00 | 1140.32 | 1451.29 | 3.13 | 10.88 | 1 | 2008 |
| FL07259-1P-4-2P-1P-M | 62.38 | 32.09 | 7.01 | 804.23 | 1787.53 | 3.23 | 14.32 | 1 | 2008 |
| FL07495-16P-2P-1P-1P-M | 61.17 | 29.94 | 6.85 | 704.50 | 1434.05 | 2.68 | 13.73 | 1 | 2008 |
| FL08335-11P-3-1P-2P-M | 63.20 | 29.86 | 6.32 | 914.19 | 1164.59 | 3.12 | 10.52 | 1 | 2009 |
| FL08480-2P-1-3P-2P-M | 58.75 | 30.22 | 6.98 | 1068.82 | 1661.71 | 3.11 | 12.65 | 1 | 2009 |
| FL09175-3P-3-3P-1P-M | 58.46 | 30.84 | 6.99 | 846.89 | 1960.05 | 2.91 | 11.94 | 1 | 2010 |
| FL09277-8P-4-1P-2P-M | 60.19 | 28.61 | 6.65 | 858.66 | 1547.69 | 3.29 | 13.44 | 1 | 2010 |
| FL09361-5P-2-2P-1P-M | 63.16 | 30.81 | 6.99 | 786.35 | 1742.94 | 3.29 | 12.02 | 1 | 2011 |
| FL10099-1P-1P-3P-2P-M | 58.50 | 31.78 | 6.84 | 1046.68 | 1649.19 | 3.20 | 11.53 | 1 | 2011 |
| FL00440-30P-12-2P-M | 61.30 | 30.10 | 5.33 | 826.77 | 1612.64 | 3.06 | 7.08 | 2 | 2000 |
| FL00447-36P-1-2P-M | 63.75 | 31.38 | 7.01 | 517.64 | 2013.09 | 2.96 | 3.78 | 2 | 2000 |
| FL00459-15P-6-1P-M | 63.40 | 29.73 | 5.82 | 803.26 | 1510.12 | 3.03 | 5.62 | 2 | 2000 |
| FL00459-27P-6-2P-M | 61.78 | 30.30 | 6.99 | 775.49 | 1938.64 | 2.97 | 6.15 | 2 | 2000 |
| FL00470-29P-11-1P-M | 62.31 | 27.06 | 6.34 | 673.64 | 1419.27 | 3.09 | 2.73 | 2 | 2000 |
| FL00477-45P-1-3P-M | 62.16 | 29.74 | 7.01 | 613.80 | 1544.43 | 2.89 | 8.34 | 2 | 2000 |
| FL00561-11P-2-3P-M | 59.80 | 29.19 | 6.99 | 625.52 | 1221.38 | 3.24 | 4.86 | 2 | 2000 |
| FL00594-1P-7-3P-M | 61.25 | 29.74 | 6.67 | 678.00 | 1288.69 | 3.12 | 6.59 | 2 | 2000 |
| FL00595-8P-2-2P-M | 58.31 | 31.01 | 7.04 | 550.65 | 1426.96 | 2.96 | 8.06 | 2 | 2000 |
| FL00826-6P-5-1P-M | 62.15 | 32.74 | 6.82 | 739.83 | 2069.90 | 3.20 | 10.21 | 2 | 2001 |
| FL00898-10P-11-1P-M | 65.43 | 29.31 | 6.51 | 938.35 | 1433.34 | 3.11 | 7.26 | 2 | 2001 |
| FL00898-17P-9-2P-M | 65.14 | 29.16 | 6.82 | 606.32 | 1433.01 | 3.17 | 2.94 | 2 | 2001 |
| FL01079-9P-1-1P-M | 61.84 | 30.54 | 7.01 | 690.48 | 1406.64 | 3.16 | 7.16 | 2 | 2001 |
| FL01870-14P-9-4P-M | 62.93 | 29.81 | 6.99 | 600.69 | 1840.32 | 3.05 | 5.35 | 2 | 2001 |
| FL01870-5P-17-1P-M | 63.54 | 28.95 | 6.66 | 704.70 | 1106.49 | 3.02 | 8.47 | 2 | 2001 |
| FL02173-3P-6-4P-2-M | 62.40 | 31.02 | 6.85 | 461.00 | 1755.45 | 3.02 | 8.06 | 2 | 2002 |
| FL03198-9P-3-1P-1P-M-1P-M | 59.99 | 32.26 | 6.99 | 774.75 | 1913.07 | 3.27 | 2.59 | 2 | 2004 |
| FL03199-24P-6-1P-1P-M | 62.58 | 31.47 | 6.99 | 802.69 | 1715.73 | 2.98 | 6.68 | 2 | 2003 |
| FL03199-24P-6-1P-2P-M | 62.11 | 31.39 | 5.97 | 706.46 | 1844.57 | 3.18 | 4.38 | 2 | 2003 |
| FL03199-26P-4-1P-3P-M | 61.80 | 30.35 | 6.83 | 765.36 | 1381.31 | 3.04 | 4.61 | 2 | 2003 |
| FL03199-2P-20-2P-2P-M | 60.75 | 32.31 | 7.01 | 794.68 | 1987.68 | 3.29 | 7.94 | 2 | 2003 |
| FL04000-5P-6-3P-3P-M | 60.68 | 31.62 | 7.02 | 652.37 | 1515.87 | 3.27 | 7.25 | 2 | 2004 |
| FL04168-3P-19-3P-3P-M | 64.07 | 31.70 | 7.00 | 1024.39 | 1827.42 | 3.07 | 5.67 | 2 | 2004 |
| FL04582-7P-2-2P-1P-M | 63.93 | 32.14 | 7.00 | 817.97 | 1902.81 | 3.04 | 10.06 | 2 | 2005 |
| FL04616-1P-9-1P-3P-M | 59.56 | 30.89 | 7.00 | 345.23 | 1801.84 | 2.75 | 8.70 | 2 | 2005 |
| FL04794-6P-4-1P-2P-M | 61.08 | 29.84 | 6.83 | 675.03 | 1242.53 | 3.12 | 6.66 | 2 | 2005 |
| FL04867-11P-3-3P-2P-M | 60.30 | 31.79 | 6.98 | 875.75 | 1669.43 | 3.09 | 8.45 | 2 | 2005 |
| FL04867-3P-18-3P-3P-M | 61.05 | 31.91 | 6.98 | 562.48 | 1610.59 | 2.96 | 2.58 | 2 | 2005 |
| FL04867-3P-18-5P-2P-M | 60.95 | 31.74 | 6.98 | 599.43 | 1582.24 | 3.12 | 4.95 | 2 | 2005 |
| FL05371-2P-2-1P-3P-M | 61.58 | 32.01 | 7.00 | 750.92 | 1708.55 | 3.21 | 5.37 | 2 | 2006 |
| FL05372-7P-3-2P-2P-M | 60.47 | 30.46 | 7.02 | 523.38 | 1611.39 | 3.15 | 4.35 | 2 | 2006 |
| FL05372-7P-4-4P-2P-M | 58.23 | 30.28 | 6.99 | 646.09 | 1762.51 | 3.14 | 7.02 | 2 | 2006 |
| FL05463-4P-13-2P-3P-M | 62.34 | 31.66 | 7.01 | 632.67 | 1825.82 | 3.06 | 6.18 | 2 | 2006 |
| FL05482-2P-19AI-1P-3P-M | 60.88 | 30.52 | 6.50 | 831.89 | 2064.42 | 2.95 | 6.95 | 2 | 2006 |
| FL05482-8P-4-2P-1P-M | 64.78 | 31.96 | 6.82 | 861.64 | 1819.85 | 3.59 | 7.68 | 2 | 2006 |
| FL05506-5P-4-2P-1P-M | 59.58 | 31.42 | 6.98 | 619.75 | 1634.19 | 3.16 | 9.09 | 2 | 2006 |
| FL06605-7P-4P-1P-3P-M | 64.96 | 32.00 | 6.35 | 895.95 | 1455.08 | 3.20 | 4.20 | 2 | 2007 |
| FL06609-21P-4-2P-3P-M | 59.36 | 30.57 | 7.00 | 522.11 | 1235.75 | 2.97 | 4.39 | 2 | 2007 |
| FL06609-21P-8-6P-3P-M | 61.49 | 30.87 | 7.00 | 778.70 | 1691.45 | 3.29 | 6.50 | 2 | 2007 |
| FL06613-20P-2AI-3P-2P-M | 59.50 | 32.57 | 6.52 | 706.24 | 1781.47 | 3.00 | 9.90 | 2 | 2007 |
| FL06733-15P-8-2P-2P-M | 62.04 | 32.75 | 6.99 | 1040.46 | 1752.23 | 2.94 | 8.49 | 2 | 2007 |
| FL06890-2P-9-2P-2P-M | 63.86 | 31.98 | 7.01 | 540.88 | 2245.90 | 3.01 | 7.07 | 2 | 2007 |
| FL06890-2P-9-2P-3P-M | 62.33 | 31.85 | 6.98 | 819.20 | 1879.20 | 3.09 | 8.54 | 2 | 2007 |
| FL06906-6P-8-2P-2P-M | 60.53 | 32.16 | 7.00 | 587.33 | 2221.04 | 2.96 | 6.41 | 2 | 2007 |
| FL07181-11P-1-3P-2P-M | 60.10 | 29.80 | 7.00 | 922.43 | 1672.48 | 3.01 | 8.17 | 2 | 2008 |
| FL07181-2P-5-5P-4P-M | 59.95 | 30.79 | 7.00 | 877.03 | 1954.21 | 2.83 | 9.32 | 2 | 2008 |
| FL07201-6P-2-2P-1P-M | 61.63 | 30.65 | 6.02 | 675.34 | 1608.05 | 3.00 | 8.27 | 2 | 2008 |
| FL07201-7P-4-1P-3P-M | 62.80 | 30.85 | 7.01 | 632.69 | 1689.06 | 3.05 | 10.04 | 2 | 2008 |
| FL07393-5P-2-1P-2P-M | 63.33 | 30.21 | 7.01 | 432.02 | 1798.35 | 2.96 | 7.43 | 2 | 2008 |
| FL07557-14P-1-2P-2P-M | 61.59 | 27.78 | 6.33 | 724.99 | 892.18 | 3.17 | 3.35 | 2 | 2008 |
| FL08389-4P-10-2P-3P-M | 60.34 | 31.45 | 6.85 | 1007.85 | 1888.61 | 3.27 | 5.86 | 2 | 2009 |
| FL08428-6P-4-2P-1P-M | 62.50 | 29.44 | 6.17 | 692.80 | 1549.68 | 3.02 | 9.58 | 2 | 2009 |
| FL09156-1P-6-1P-2P-M | 60.89 | 29.82 | 7.01 | 824.84 | 1556.76 | 3.09 | 5.74 | 2 | 2010 |
| FL09201-5P-10-3P-3P-M | 60.71 | 29.48 | 6.67 | 777.13 | 1592.75 | 3.18 | 4.89 | 2 | 2010 |
| FL09206-4P-2-1P-1P-M | 60.86 | 30.59 | 7.00 | 910.50 | 1654.49 | 3.40 | 10.41 | 2 | 2010 |
| FL09292-26P-1-2P-1P-M | 62.98 | 30.36 | 6.85 | 842.31 | 1022.88 | 3.18 | 9.39 | 2 | 2011 |
| FL09369-3P-3-3P-2P-M | 60.29 | 30.05 | 6.98 | 596.13 | 1726.19 | 3.07 | 6.72 | 2 | 2010 |
| FL09378-12P-3-2P-2P-M | 58.81 | 30.92 | 6.36 | 648.03 | 2054.54 | 3.07 | 4.15 | 2 | 2010 |
| FL09378-5P-2-2P-1P-M | 60.02 | 31.84 | 7.01 | 366.38 | 1885.04 | 2.93 | 2.01 | 2 | 2010 |
| FL09401-3P-3-4P-1P-M | 59.49 | 29.31 | 6.33 | 703.19 | 1766.30 | 2.98 | 4.40 | 2 | 2010 |
| FL09407-13P-3-5P-1P-M | 59.66 | 28.42 | 7.01 | 581.38 | 2117.60 | 3.10 | 9.16 | 2 | 2010 |
| FL09504-6P-7-1P-2P-M | 62.19 | 31.41 | 7.00 | 750.93 | 1929.66 | 3.17 | 7.50 | 2 | 2010 |
| FL09508-1P-3-1P-2P-M | 63.61 | 32.10 | 6.99 | 768.96 | 1947.58 | 3.08 | 4.04 | 2 | 2010 |
| FL09525-2P-7-1P-3P-M | 59.77 | 30.45 | 6.48 | 689.72 | 1758.19 | 3.01 | 2.19 | 2 | 2011 |
| FL09554-6P-2-3P-1P-M | 59.73 | 30.20 | 6.99 | 736.30 | 1693.73 | 2.87 | 7.85 | 2 | 2010 |
| FL09657-2P-6-1P-1P-M | 59.92 | 28.72 | 6.49 | 804.25 | 1129.99 | 3.27 | 6.88 | 2 | 2012 |
| FL10134-16P-4-3P-1P-M | 62.06 | 32.02 | 7.00 | 803.26 | 1886.37 | 3.41 | 10.41 | 2 | 2011 |
| FL10381-4P-2-1P-1P-M | 59.80 | 30.16 | 7.00 | 850.81 | 1928.48 | 2.94 | 6.63 | 2 | 2011 |
| FL10384-4P-2-2P-2P-M | 62.59 | 30.68 | 7.00 | 813.73 | 1702.35 | 3.50 | 6.57 | 2 | 2011 |
| FL10961-1P-1-3P-3P-M | 59.22 | 28.93 | 6.83 | 580.62 | 965.61 | 3.07 | 5.01 | 2 | 2012 |
| FL11044-8P-3-1P-1P-M | 60.27 | 30.15 | 6.99 | 1168.52 | 1421.95 | 3.14 | 6.40 | 2 | 2012 |
| FL11094-4P-3-2P-3P-M | 58.97 | 29.27 | 7.01 | 943.47 | 1373.78 | 3.25 | 6.07 | 2 | 2012 |
| FL11175-2P-2-2P-2P-M | 58.86 | 31.15 | 6.84 | 763.93 | 1743.83 | 3.21 | 8.15 | 2 | 2012 |
| FL11175-4P-3-2P-2P-M | 60.19 | 30.58 | 7.00 | 932.33 | 1137.71 | 3.22 | 6.90 | 2 | 2012 |
| FL11231-3P-5-2P-1P-M | 61.55 | 30.93 | 6.16 | 609.59 | 1722.90 | 2.98 | 3.97 | 2 | 2012 |
| FL11424-1P-1-3P-3P-M | 62.94 | 29.50 | 7.00 | 458.41 | 1764.91 | 3.11 | 5.16 | 2 | 2013 |
| FL11434-2P-4-2P-2P-M | 59.72 | 31.81 | 7.01 | 651.22 | 2018.88 | 3.34 | 6.01 | 2 | 2013 |
| FL12017-2P-3-1P-2P-M | 60.75 | 30.41 | 6.83 | 1006.40 | 1874.62 | 3.16 | 7.17 | 2 | 2013 |
| FL13032-4P-5SR-1P-2P-M-M | 63.26 | 29.93 | 6.67 | 716.94 | 1368.38 | 3.21 | 4.81 | 2 | 2014 |
| FL13032-6P-2SR-1P-2P-M-M | 62.13 | 30.12 | 6.66 | 549.67 | 1636.76 | 3.35 | 5.74 | 2 | 2014 |
| FL13299-1P-5SR-3P-2P-M-M | 64.81 | 29.59 | 6.32 | 946.00 | 1525.14 | 2.97 | 9.21 | 2 | 2014 |
| FL13328-3P-3SR-3P-3P-M-M | 61.52 | 30.88 | 6.19 | 654.52 | 1133.39 | 3.33 | 8.44 | 2 | 2014 |
| FL13379-1P-3SR-2P-2P-M-MP | 63.54 | 31.96 | 7.00 | 751.35 | 1967.28 | 3.09 | 4.36 | 2 | 2015 |
| FL13379-1P-3SR-2P-3P-M-MP | 62.95 | 31.77 | 7.03 | 525.74 | 1876.38 | 3.14 | 4.90 | 2 | 2015 |
| FL13848-3P-3SR-3P-3P-M-MP | 61.35 | 32.32 | 6.99 | 735.32 | 1559.33 | 3.32 | 9.54 | 2 | 2015 |
| FL14144-6P-1P-3P-1P-M-MP | 61.21 | 31.16 | 6.83 | 912.77 | 1586.08 | 3.08 | 5.97 | 2 | 2015 |
| FL14194-3P-8SR-2P-2P-M-MP | 62.35 | 30.87 | 7.01 | 576.24 | 1469.30 | 3.24 | 4.77 | 2 | 2015 |
| FL14200-1P-2SR-3P-1P-M-MP | 60.44 | 29.86 | 6.67 | 892.36 | 1175.69 | 3.19 | 4.94 | 2 | 2015 |
| FL14223-6P-4SR-3P-3P-M-MP | 59.16 | 30.53 | 6.98 | 563.49 | 1723.02 | 3.06 | 5.65 | 2 | 2015 |
| FL00138-3P-7-11P-M | 50.65 | 29.92 | 5.00 | 992.20 | 1892.63 | 3.47 | 10.33 | 3 | 1999 |
| FL00147-8P-15-3P-M | 55.64 | 31.69 | 7.00 | 498.31 | 1472.49 | 3.49 | 12.58 | 3 | 1999 |
| FL00972-1P-4-2P-M | 57.51 | 29.74 | 5.33 | 776.85 | 1593.75 | 3.38 | 14.54 | 3 | 2001 |
| FL01841-21P-4-2P-M | 57.05 | 28.99 | 6.98 | 661.16 | 1207.29 | 3.13 | 6.91 | 3 | 2001 |
| FL02066-4P-1-1P-2-M | 57.64 | 29.82 | 7.00 | 700.87 | 1214.47 | 3.05 | 9.13 | 3 | 2002 |
| FL02173-1P-3-2P-2P-M | 51.84 | 31.71 | 6.83 | 412.98 | 878.32 | 3.07 | 19.29 | 3 | 2002 |
| FL02755-1P-2-3P-1-M | 55.74 | 31.61 | 7.00 | 449.48 | 1113.12 | 2.99 | 10.77 | 3 | 2002 |
| FL02755-1P-2-7P-2-M | 57.94 | 31.79 | 6.84 | 254.94 | 871.83 | 3.26 | 14.00 | 3 | 2002 |
| FL03160-6P-9-2P-2P-M | 62.11 | 31.38 | 7.00 | 269.54 | 684.25 | 3.38 | 6.28 | 3 | 2003 |
| FL03191-5P-1-3P-1P-M | 53.21 | 31.85 | 7.01 | 623.97 | 1904.57 | 3.48 | 2.00 | 3 | 2003 |
| FL03194-8P-2-1P-2P-M | 55.70 | 32.42 | 7.01 | 625.33 | 1563.70 | 3.19 | 16.86 | 3 | 2003 |
| FL04676-3P-4-1P-2P-M | 59.10 | 31.59 | 7.03 | 383.66 | 811.22 | 3.27 | 7.86 | 3 | 2005 |
| FL04837-8P-1-2P-3P-M | 55.18 | 29.74 | 7.00 | 581.27 | 715.62 | 3.21 | 11.87 | 3 | 2005 |
| FL04837-8P-10-1P-1P-M | 55.03 | 31.36 | 7.00 | 559.93 | 1378.65 | 3.08 | 10.41 | 3 | 2005 |
| FL04837-8P-9-1P-1P-M | 51.75 | 30.94 | 6.34 | 721.91 | 1270.37 | 3.12 | 11.39 | 3 | 2005 |
| FL04867-3P-4-3P-2P-M | 58.03 | 31.96 | 7.00 | 396.88 | 1089.28 | 3.17 | 3.72 | 3 | 2005 |
| FL04904-8P-8-1P-2P-M | 56.80 | 30.81 | 7.01 | 156.92 | 751.89 | 3.26 | 6.67 | 3 | 2005 |
| FL05496-5P-6-3P-1P-M | 57.08 | 32.13 | 7.01 | 573.72 | 668.76 | 3.33 | 1.98 | 3 | 2006 |
| FL05496-5P-6-3P-3P-M | 52.64 | 31.24 | 6.16 | 497.44 | 855.49 | 3.26 | 7.48 | 3 | 2006 |
| FL05598-5P-3-2P-3P-M | 58.70 | 31.03 | 7.00 | 553.79 | 1487.26 | 3.26 | 5.58 | 3 | 2006 |
| FL06710-5P-14-7P-3P-M | 56.71 | 31.81 | 6.99 | 618.97 | 1465.43 | 3.43 | 11.69 | 3 | 2007 |
| FL06906-13P-2-3P-2P-M | 54.12 | 31.80 | 7.00 | 880.74 | 1396.22 | 3.31 | 14.91 | 3 | 2007 |
| FL07370-16P-1-2P-1P-M | 59.28 | 32.19 | 7.01 | 711.32 | 1485.83 | 3.51 | 6.00 | 3 | 2008 |
| FL07395-2P-2-3P-1P-M | 58.78 | 31.50 | 6.16 | 756.66 | 1859.15 | 3.38 | 6.88 | 3 | 2008 |
| FL08190-3P-11-4P-1P-M | 56.69 | 30.66 | 6.98 | 522.26 | 1232.79 | 3.50 | 10.62 | 3 | 2009 |
| FL08215-7P-4-3P-2P-M | 56.86 | 29.89 | 6.84 | 696.15 | 1049.32 | 3.27 | 11.84 | 3 | 2009 |
| FL08224-1P-5-2P-2P-M | 58.34 | 31.18 | 5.63 | 685.16 | 1344.73 | 3.54 | 5.64 | 3 | 2009 |
| FL08417-18P-2-2P-2P-M | 56.78 | 29.92 | 5.00 | 832.82 | 1606.05 | 3.60 | 9.20 | 3 | 2009 |
| FL09203-10P-2-1P-3P-M | 57.77 | 29.34 | 6.82 | 635.96 | 703.80 | 3.16 | 5.42 | 3 | 2010 |
| FL09474-12P-7-1P-2P-M | 57.95 | 29.70 | 6.82 | 599.32 | 1676.50 | 3.29 | 3.90 | 3 | 2010 |
| FL09477-10P-3-3P-3P-M | 61.48 | 29.74 | 6.83 | 776.79 | 1488.35 | 3.52 | 10.13 | 3 | 2010 |
| FL09480-12P-4-2P-4P-M | 58.69 | 32.16 | 7.01 | 674.73 | 1861.05 | 3.71 | 8.89 | 3 | 2010 |
| FL09482-6P-5-2P-2P-M | 60.08 | 30.32 | 7.04 | 766.08 | 1567.81 | 3.45 | 5.82 | 3 | 2010 |
| FL09581-2P-3-1P-2P-M | 58.52 | 30.53 | 7.01 | 721.74 | 1586.84 | 3.30 | 3.56 | 3 | 2011 |
| FL10030-5P-4-2P-1P-M | 58.39 | 31.85 | 6.34 | 564.21 | 1280.03 | 3.41 | 5.36 | 3 | 2011 |
| FL10031-2P-12-1P-1P-M | 57.48 | 31.51 | 6.98 | 425.53 | 1005.51 | 2.96 | 11.40 | 3 | 2011 |
| FL10031-4P-4-1P-2P-M | 55.87 | 30.67 | 6.66 | 551.80 | 935.58 | 3.25 | 6.98 | 3 | 2011 |
| FL10031-4P-4-2P-1P-M | 55.86 | 30.60 | 6.99 | 297.22 | 727.74 | 3.19 | 3.85 | 3 | 2011 |
| FL10059-2P-3-3P-3P-M | 58.17 | 30.87 | 6.84 | 514.54 | 1400.94 | 3.86 | 4.39 | 3 | 2011 |
| FL10081-25P-1P-1P-1P-M | 57.98 | 29.88 | 6.99 | 587.12 | 1130.12 | 3.62 | 10.98 | 3 | 2011 |
| FL10100-13P-4-1P-2P-M | 56.12 | 32.04 | 6.99 | 571.00 | 2116.17 | 3.60 | 14.32 | 3 | 2011 |
| FL10391-7P-1-2P-1P-M | 56.98 | 29.54 | 6.35 | 821.48 | 1345.60 | 3.42 | 9.52 | 3 | 2011 |
| FL10391-7P-1-3P-2P-M | 56.52 | 30.12 | 5.19 | 883.15 | 1479.26 | 3.36 | 11.45 | 3 | 2011 |
| FL10395-7P-1-3P-3P-M | 58.55 | 31.71 | 7.01 | 570.92 | 1519.03 | 3.21 | 9.55 | 3 | 2011 |
| FL10893-6P-6-2P-1P-M | 54.74 | 30.07 | 6.17 | 945.32 | 1387.48 | 3.32 | 11.41 | 3 | 2012 |
| FL10978-3P-6-1P-3P-M | 59.87 | 30.98 | 7.00 | 691.91 | 1978.18 | 3.55 | 5.52 | 3 | 2012 |
| FL11097-3P-1-2P-1P-M | 61.12 | 30.13 | 6.32 | 580.00 | 892.19 | 3.32 | 10.94 | 3 | 2012 |
| FL11231-3P-7-3P-2P-M | 56.13 | 31.03 | 6.51 | 872.24 | 1526.75 | 3.51 | 6.62 | 3 | 2012 |
| FL11294-1P-9-1P-2P-M | 55.70 | 27.73 | 5.35 | 769.94 | 1063.44 | 3.63 | 11.93 | 3 | 2012 |
| FL11319-1P-3-2P-3P-M | 53.28 | 30.84 | 7.00 | 782.76 | 1525.41 | 3.39 | 7.42 | 3 | 2013 |
| FL11338-7P-5-3P-1P-M | 56.10 | 29.85 | 6.99 | 450.85 | 715.48 | 3.24 | 9.31 | 3 | 2013 |
| FL11391-3P-2-2P-3P-M | 57.66 | 31.25 | 6.99 | 587.89 | 1737.01 | 3.32 | 5.39 | 3 | 2013 |
| FL11999-1P-2-1P-2P-M | 53.00 | 29.62 | 6.15 | 777.73 | 1461.02 | 3.43 | 5.35 | 3 | 2013 |
| FL12086-2P-4-2P-1P-M | 58.06 | 31.70 | 7.01 | 849.84 | 1730.83 | 3.58 | 7.15 | 3 | 2013 |
| FL13065-8P-1P-1P-1P-M-M | 59.84 | 31.16 | 7.02 | 761.77 | 1835.18 | 3.44 | 13.53 | 3 | 2014 |
| FL13102-1P-1SR-3P-1P-M-M | 57.81 | 29.12 | 6.99 | 793.17 | 1319.00 | 3.32 | 11.83 | 3 | 2014 |
| FL13109-1P-8SR-3P-1P-M-M | 54.70 | 30.52 | 7.01 | 1018.31 | 2136.36 | 3.94 | 12.65 | 3 | 2014 |
| FL13210-1P-3SR-3P-1P-M-M | 54.11 | 30.21 | 6.84 | 600.95 | 1447.08 | 3.48 | 4.23 | 3 | 2014 |
| FL13900-2P-4SR-3P-1P-M-MP | 55.90 | 31.38 | 7.00 | 859.00 | 1718.00 | 3.70 | 5.13 | 3 | 2015 |
| FL14145-2P-1SR-2P-3P-M-MP | 57.00 | 30.98 | 7.00 | 813.75 | 1838.09 | 3.38 | 3.67 | 3 | 2015 |
| FL14212-3P-4SR-2P-3P-M-MP | 54.30 | 31.62 | 6.99 | 529.37 | 1885.52 | 3.68 | 9.54 | 3 | 2015 |
| FL14217-4P-1SR-2P-2P-M-MP | 58.48 | 31.56 | 7.02 | 688.75 | 1698.76 | 3.47 | 9.69 | 3 | 2015 |
| FL00138-3P-7-9P-M | 59.88 | 29.48 | 2.18 | 751.51 | 1859.17 | 3.57 | 11.02 | 4 | 1999 |
| FL00147-10P-9-4P-M | 57.92 | 28.47 | 4.00 | 1109.70 | 1081.47 | 3.19 | 8.93 | 4 | 1999 |
| FL00147-1P-5-15P-M | 55.52 | 29.90 | 3.52 | 1131.05 | 1484.65 | 3.25 | 11.43 | 4 | 1999 |
| FL00147-2P-13-9P-M | 59.18 | 29.34 | 2.83 | 811.15 | 1728.88 | 3.21 | 5.96 | 4 | 1999 |
| FL00147-8P-8-11P-M | 51.47 | 29.77 | 2.17 | 864.32 | 1001.51 | 3.62 | 15.94 | 4 | 1999 |
| FL00159-6P-2-12P-M | 63.82 | 28.43 | 4.82 | 1173.78 | 995.75 | 3.15 | 7.93 | 4 | 1999 |
| FL00836-13P-3-1P-M | 58.73 | 28.77 | 4.99 | 1038.42 | 1156.12 | 2.87 | 13.59 | 4 | 2001 |
| FL01911-12P-5-1P-M | 59.15 | 28.98 | 2.35 | 643.54 | 575.70 | 3.10 | 18.58 | 4 | 2001 |
| FL02124-2P-5-1P-1-M | 58.53 | 28.31 | 4.99 | 1351.61 | 521.57 | 3.14 | 15.30 | 4 | 2002 |
| FL02173-1P-8-1P-3-M | 55.17 | 30.96 | 4.82 | 995.85 | 1409.18 | 3.16 | 12.40 | 4 | 2002 |
| FL03157-10P-6-1P-1P-M | 64.11 | 31.04 | 2.34 | 716.05 | 1611.51 | 3.19 | 4.15 | 4 | 2003 |
| FL03186-1P-19-2P-1P-M | 61.32 | 25.89 | 3.50 | 1053.58 | 825.24 | 3.18 | 7.83 | 4 | 2003 |
| FL03188-7P-1-1P-3P-M | 50.58 | 30.85 | 4.52 | 1119.93 | 1568.26 | 3.31 | 9.08 | 4 | 2003 |
| FL03188-7P-1-2P-3P-M | 55.62 | 28.59 | 4.01 | 1220.60 | 1095.40 | 3.08 | 8.98 | 4 | 2003 |
| FL03191-4P-48-2P-2P-M | 57.31 | 29.71 | 2.83 | 1083.16 | 1109.83 | 3.07 | 17.06 | 4 | 2003 |
| FL03191-4P-57-2P-1P-M | 58.32 | 30.46 | 2.84 | 1167.36 | 1275.45 | 3.32 | 16.49 | 4 | 2003 |
| FL03191-5P-2-2P-1P-M | 55.71 | 30.96 | 3.18 | 1069.36 | 1453.53 | 3.69 | 7.02 | 4 | 2003 |
| FL03710-1P-7-1P-3P-M | 57.74 | 28.98 | 2.67 | 1101.70 | 864.05 | 2.83 | 24.07 | 4 | 2004 |
| FL03710-5P-4-2P-1P-M | 58.52 | 28.59 | 5.82 | 1098.62 | 1436.22 | 3.04 | 9.70 | 4 | 2004 |
| FL03710-8P-10-2P-2P-M | 56.89 | 30.18 | 4.00 | 991.48 | 1289.23 | 3.01 | 14.50 | 4 | 2004 |
| FL03724-11P-26-4P-3P-M | 62.85 | 30.78 | 3.82 | 1160.60 | 1182.68 | 3.07 | 22.40 | 4 | 2004 |
| FL03724-3P-12-1P-2P-M | 62.80 | 29.50 | 3.03 | 1216.83 | 1034.29 | 2.86 | 10.32 | 4 | 2004 |
| FL03779-4P-10-4P-2P-M | 59.47 | 30.01 | 3.34 | 1213.63 | 1051.68 | 3.32 | 25.28 | 4 | 2004 |
| FL04676-1P-4-1P-1P-M | 63.25 | 29.98 | 3.01 | 1166.24 | 1158.70 | 3.18 | 11.68 | 4 | 2005 |
| FL04894-3P-28-1P-2P-M | 53.32 | 30.25 | 4.35 | 806.52 | 1210.91 | 3.08 | 19.51 | 4 | 2005 |
| FL05392-3P-14-1P-1P-M | 59.76 | 30.38 | 3.67 | 1251.26 | 1028.01 | 2.83 | 28.08 | 4 | 2006 |
| FL05400-8P-6-2P-3P-M | 60.27 | 29.84 | 2.51 | 1073.62 | 969.33 | 3.00 | 19.88 | 4 | 2006 |
| FL05463-1P-13-2P-2P-M | 61.30 | 30.51 | 4.33 | 960.69 | 1535.44 | 3.30 | 11.67 | 4 | 2006 |
| FL05464-6P-16-3P-3P-M | 61.62 | 31.06 | 3.35 | 964.03 | 1364.95 | 3.24 | 7.35 | 4 | 2006 |
| FL05658-7P-1-4P-1P-M | 62.37 | 29.25 | 4.15 | 914.51 | 1746.44 | 3.07 | 5.46 | 4 | 2006 |
| FL05658-7P-1-5P-3P-M | 63.27 | 30.40 | 3.99 | 1050.10 | 1353.63 | 2.86 | 17.09 | 4 | 2006 |
| FL06562-6P-2-1P-2P-M | 52.12 | 29.72 | 3.67 | 935.99 | 1330.87 | 2.95 | 8.73 | 4 | 2007 |
| FL06608-6P-5-4P-2P-M | 58.39 | 29.71 | 4.21 | 1138.43 | 1341.98 | 2.97 | 7.24 | 4 | 2007 |
| FL07474-2P-2-2P-3P-M | 58.09 | 28.61 | 3.00 | 784.39 | 1758.65 | 3.11 | 11.07 | 4 | 2008 |
| FL07513-3P-4-4P-1P-M | 62.31 | 28.88 | 3.99 | 909.55 | 1537.81 | 3.23 | 4.47 | 4 | 2008 |
| FL08219-1P-8-1P-1P-M | 57.59 | 27.94 | 3.66 | 711.24 | 1298.69 | 3.05 | 8.92 | 4 | 2009 |
| FL08219-1P-8-3P-1P-M | 54.76 | 29.02 | 2.84 | 752.33 | 1356.09 | 3.25 | 16.22 | 4 | 2009 |
| FL08417-16P-2-1P-3P-M | 58.52 | 29.33 | 3.50 | 991.99 | 1517.43 | 3.43 | 15.15 | 4 | 2009 |
| FL09252-2P-14-3P-1P-M | 61.69 | 30.20 | 4.33 | 669.46 | 1628.82 | 3.05 | 12.34 | 4 | 2010 |
| FL09389-3P-3P-1P-3P-M | 57.64 | 28.77 | 2.33 | 848.57 | 1543.88 | 3.12 | 8.13 | 4 | 2010 |
| FL10384-2P-4-2P-3P-M | 62.57 | 28.62 | 3.84 | 879.74 | 1704.20 | 3.15 | 4.76 | 4 | 2011 |
| FL10417-12P-4-2P-3P-M | 57.89 | 28.84 | 1.82 | 630.43 | 1925.09 | 3.11 | 11.45 | 4 | 2012 |
| FL10915-3P-3-1P-3P-M | 59.07 | 27.55 | 2.32 | 964.94 | 1172.38 | 3.45 | 2.61 | 4 | 2012 |
| FL11066-9P-5P-3P-1P-M | 61.70 | 30.39 | 4.67 | 1000.83 | 1146.06 | 3.30 | 15.25 | 4 | 2012 |
| FL11444-3P-8-3P-2P-M | 53.70 | 28.70 | 3.98 | 700.99 | 2164.05 | 3.19 | 6.21 | 4 | 2013 |
| FL11963-10P-7-2P-1P-M | 59.02 | 28.16 | 3.17 | 1112.16 | 752.81 | 3.35 | 12.50 | 4 | 2013 |
| FL12156-3P-9-3P-2P-M | 54.97 | 28.22 | 2.69 | 1162.76 | 910.16 | 3.20 | 8.98 | 4 | 2013 |
| FL13303-1P-1SR-2P-3P-M-M | 55.04 | 27.59 | 2.49 | 1237.42 | 552.64 | 2.96 | 25.63 | 4 | 2014 |
| FL13309-1P-5SR-2P-3P-M-M | 59.56 | 29.59 | 3.99 | 1089.77 | 1313.20 | 2.83 | 9.60 | 4 | 2014 |
| FL14164-5P-3SR-1P-3P-M-MP | 59.47 | 28.75 | 3.50 | 971.84 | 1107.86 | 3.56 | 6.46 | 4 | 2015 |
| FL001028-8P-3-1P-1P-1-M | 61.28 | 25.75 | 5.97 | 1069.87 | 909.76 | 3.01 | 6.62 | 5 | 2002 |
| FL00470-23P-3-2P-M | 63.90 | 25.29 | 6.32 | 1473.81 | 136.37 | 2.97 | 3.90 | 5 | 2000 |
| FL00518-12P-4-2P-M | 60.57 | 24.38 | 6.17 | 1453.40 | -35.33 | 3.13 | 5.54 | 5 | 2000 |
| FL00593-6P-1-3P-M | 62.98 | 24.20 | 6.32 | 1373.23 | 22.14 | 3.19 | 3.21 | 5 | 2000 |
| FL00855-1P-2-2P-M | 59.84 | 25.91 | 5.34 | 1003.15 | 922.38 | 3.00 | 6.72 | 5 | 2001 |
| FL00984-10P-5-2P-M | 61.85 | 24.84 | 5.81 | 1603.71 | -24.79 | 3.14 | 6.74 | 5 | 2001 |
| FL02063-4P-1-4P-1-M | 63.48 | 27.93 | 6.53 | 1049.66 | 726.79 | 3.22 | 4.82 | 5 | 2002 |
| FL03001-MP-2-1P-3P-M | 58.65 | 28.48 | 6.50 | 1001.02 | 972.58 | 3.05 | 3.22 | 5 | 2003 |
| FL03232-30P-8-3P-1P-M-1P-M | 59.74 | 23.36 | 6.16 | 1499.20 | -135.30 | 3.15 | 3.20 | 5 | 2004 |
| FL03233-8P-6-2P-2P-M | 59.96 | 28.02 | 6.52 | 1094.78 | 890.38 | 3.34 | 2.39 | 5 | 2003 |
| FL03233-8P-6-2P-5P-M-1P-M | 61.89 | 27.29 | 5.98 | 1115.21 | 781.42 | 3.25 | 3.35 | 5 | 2004 |
| FL03273-3P-15-3P-2P-M | 62.22 | 24.44 | 5.17 | 1485.38 | -242.97 | 3.29 | 4.55 | 5 | 2003 |
| FL03273-4P-7-2P-3P-M | 60.80 | 24.05 | 6.15 | 1419.36 | 159.66 | 3.22 | 5.66 | 5 | 2003 |
| FL03746-9P-4-2P-2P-M | 59.96 | 25.71 | 5.99 | 1068.34 | 526.37 | 3.06 | 2.40 | 5 | 2004 |
| FL04585-13P-4-2P-2P-M | 61.87 | 26.86 | 6.16 | 1214.20 | 570.49 | 3.25 | 2.99 | 5 | 2005 |
| FL06609-19P-2P-4P-2P-M | 59.04 | 24.33 | 6.16 | 1570.01 | -89.85 | 3.45 | 4.40 | 5 | 2007 |
| FL07157-3P-5-3P-1P-M | 56.79 | 25.49 | 6.02 | 1717.07 | -213.17 | 3.84 | 6.72 | 5 | 2008 |
| FL07173-1P-11-1P-3P-M | 49.58 | 25.51 | 5.98 | 1243.45 | 322.59 | 4.09 | 3.72 | 5 | 2008 |
| FL07173-1P-5-3P-3P-M | 49.16 | 26.86 | 6.50 | 1171.25 | 577.24 | 4.09 | 2.18 | 5 | 2008 |
| FL07334-10P-3-3P-1P-M | 60.77 | 24.91 | 6.18 | 1561.07 | 218.30 | 3.12 | 6.56 | 5 | 2008 |
| FL08247-1P-9-1P-4P-M | 61.72 | 25.04 | 6.02 | 1357.92 | 289.20 | 3.12 | 4.84 | 5 | 2009 |
| FL08269-2P-3-2P-1P-M | 63.88 | 25.35 | 6.16 | 1191.21 | 391.10 | 3.42 | 5.89 | 5 | 2009 |
| FL08269-2P-3-2P-2P-M | 60.90 | 24.85 | 6.00 | 1204.04 | 334.73 | 3.44 | 4.33 | 5 | 2009 |
| FL08310-4P-1-3P-3P-M | 56.88 | 26.07 | 6.15 | 1282.55 | 611.01 | 2.96 | 1.90 | 5 | 2009 |
| FL08314-2P-4-1P-2P-M | 60.41 | 25.54 | 6.32 | 983.46 | 688.21 | 3.00 | 2.16 | 5 | 2009 |
| FL08327-2P-2-3P-2P-M | 59.52 | 27.28 | 5.68 | 1277.95 | 394.34 | 3.24 | 8.08 | 5 | 2009 |
| FL08378-3P-5-2P-4P-M | 60.19 | 28.16 | 6.18 | 1196.43 | 746.05 | 3.36 | 3.32 | 5 | 2009 |
| FL10381-4P-2-2P-4P-M | 56.74 | 26.48 | 6.17 | 1077.77 | 595.07 | 3.44 | 6.13 | 5 | 2011 |
| FL12031-6P-1-3P-1P-M | 56.61 | 27.04 | 6.17 | 1100.98 | 641.72 | 3.31 | 4.27 | 5 | 2013 |
| FL12175-6P-6SR-1P-2P-M-M | 61.17 | 25.65 | 6.00 | 1378.24 | 288.82 | 3.32 | 3.87 | 5 | 2014 |
| FL12239-1P-1P-3P-3P-M | 51.16 | 23.45 | 5.67 | 1191.23 | 476.89 | 3.58 | 2.44 | 5 | 2013 |
| FL12263-6P-5SR-2P-2P-M-M | 54.93 | 27.17 | 6.49 | 1228.47 | 597.18 | 3.39 | 8.82 | 5 | 2014 |
| FL13032-3P-1SR-3P-2P-M-M | 55.44 | 25.98 | 6.32 | 1061.62 | 585.66 | 3.45 | 2.63 | 5 | 2014 |
| FL13192-7P-4SR-3P-3P-M-M | 62.99 | 28.09 | 4.47 | 1012.33 | 765.22 | 3.27 | 4.70 | 5 | 2014 |
| FL14188-5P-2SR-2P-3P-M-MP | 60.98 | 26.94 | 6.17 | 1143.49 | 522.81 | 3.32 | 1.60 | 5 | 2015 |
| FL14215-3P-6SR-2P-2P-M-MP | 62.11 | 27.24 | 6.16 | 1120.37 | 737.82 | 3.12 | 4.59 | 5 | 2015 |

**A**

**B**

**C**

**Supplemental Figure 4**. PCA of traits $OHRR$, $AC$, $GT$, $BDv$, $SBv$, $GLWR$, and $WCRK$ evaluated in the VIP color coded by each group.

**Supplemental Figure 5.** Grain quality Genome Wide Association Studies (GWAS). GWA Manhattan and Q-Q plots for **A)** optimal-head-rice-recovery ($OHRR$) and **B)** delayed-head-rice-recovery ($DHRR$).

**Supplemental Figure 5.** Grain quality Genome Wide Association Studies (GWAS). GWA Manhattan and Q-Q plots for **C)** amylose-content ($AC$) and **D)** gelatinization-temperature expressed as alkali spread value ($GT-ASV$).

**Supplemental Figure 5.** Grain quality Genome Wide Association Studies (GWAS). GWA Manhattan and Q-Q plots for **E)** breakdown-viscosity ($BDv$) and **F)** setback-viscosity ($SBv$).

**Supplemental Figure 5.** Grain quality Genome Wide Association Studies (GWAS). GWA Manhattan and Q-Q plots for **G)** grain-length-to-width-ratio ($GLWR$) and **H)** white-core-rice-kernel ($WCRK$).

**Supplemental Table 4**. Genomic selection cross validations analysis for 10 grain quality traits using four GS models, ridge regression (*rrBLUP*), reproducing kernel Hilbert space (*RKHS*), Bayesian LASSO (*BL*) and random forest (*RF*).

| Trait | Model | Predictive ability | CI lower | CI upper |
| --- | --- | --- | --- | --- |
| *OHRR* | *rrBLUP* | 0.427 | 0.375 | 0.479 |
| *OHRR* | *RKHS* | 0.429 | 0.377 | 0.481 |
| *OHRR* | *BL* | 0.397 | 0.345 | 0.449 |
| *OHRR* | *RF* | 0.458 | 0.406 | 0.510 |
| *DHRR* | *rrBLUP* | 0.427 | 0.375 | 0.479 |
| *DHRR* | *RKHS* | 0.429 | 0.377 | 0.481 |
| *DHRR* | *BL* | 0.469 | 0.417 | 0.521 |
| *DHRR* | *RF* | 0.446 | 0.394 | 0.498 |
| *AC* | *rrBLUP* | 0.319 | 0.267 | 0.371 |
| *AC* | *RKHS* | 0.325 | 0.273 | 0.377 |
| *AC* | *BL* | 0.549 | 0.497 | 0.601 |
| *AC* | *RF* | 0.619 | 0.567 | 0.671 |
| *GT-ASV* | *rrBLUP* | 0.485 | 0.433 | 0.537 |
| *GT-ASV* | *RKHS* | 0.487 | 0.435 | 0.539 |
| *GT-ASV* | *BL* | 0.501 | 0.449 | 0.553 |
| *GT-ASV* | *RF* | 0.481 | 0.429 | 0.533 |
| *BDv* | *rrBLUP* | 0.371 | 0.319 | 0.423 |
| *BDv* | *RKHS* | 0.371 | 0.319 | 0.423 |
| *BDv* | *BL* | 0.406 | 0.354 | 0.458 |
| *BDv* | *RF* | 0.484 | 0.432 | 0.536 |
| *SBv* | *rrBLUP* | 0.395 | 0.343 | 0.447 |
| *SBv* | *RKHS* | 0.395 | 0.343 | 0.447 |
| *SBv* | *BL* | 0.686 | 0.634 | 0.738 |
| *SBv* | *RF* | 0.723 | 0.671 | 0.775 |
| *GL* | *rrBLUP* | 0.527 | 0.475 | 0.579 |
| *GL* | *RKHS* | 0.527 | 0.475 | 0.579 |
| *GL* | *BL* | 0.568 | 0.516 | 0.620 |
| *GL* | *RF* | 0.538 | 0.486 | 0.590 |
| *GW* | *rrBLUP* | 0.436 | 0.384 | 0.488 |
| *GW* | *RKHS* | 0.429 | 0.377 | 0.481 |
| *GW* | *BL* | 0.511 | 0.459 | 0.563 |
| *GW* | *RF* | 0.448 | 0.396 | 0.500 |
| *GLWR* | *rrBLUP* | 0.543 | 0.491 | 0.595 |
| *GLWR* | *RKHS* | 0.544 | 0.492 | 0.596 |
| *GLWR* | *BL* | 0.635 | 0.583 | 0.687 |
| *GLWR* | *RF* | 0.595 | 0.543 | 0.647 |
| *WCRK* | *rrBLUP* | 0.457 | 0.405 | 0.509 |
| *WCRK* | *RKHS* | 0.458 | 0.406 | 0.510 |
| *WCRK* | *BL* | 0.465 | 0.413 | 0.517 |
| *WCRK* | *RF* | 0.477 | 0.425 | 0.529 |

**Supplemental Table 5**	. **Haplotype analysis for** $\boldsymbol{AC}$**,** $\boldsymbol{GT-ASV}$**, and** $\boldsymbol{SBv}$**.**

| Markers | Haplotype | Adj. means | SD | Tukey test | Trait |
| --- | --- | --- | --- | --- | --- |
| WX-INT//WX-A_GROUP//WX-A-RC222 | AA_CC_CC | 30.674 | 1.59 | a | **AC** |
| WX-INT//WX-A_GROUP//WX-A-RC222 | AA_CC_TT | 30.543 | 1.12 | a | **AC** |
| WX-INT//WX-A_GROUP//WX-A-RC222 | CC_TT_CC | 29.606 | 1.26 | b | **AC** |
| WX-INT//WX-A_GROUP//WX-A-RC222 | CC_CC_CC | 29.383 | 1.13 | b | **AC** |
| WX-INT//WX-A_GROUP//WX-A-RC222 | AA_TT_CC | 26.099 | 1.58 | c | **AC** |
| WX-A_GROUP//WX-A-RC222 | CC_TT | 1585.81 | 301.13 | a | **SBv** |
| WX-A_GROUP//WX-A-RC222 | CC_CC | 1009.64 | 319.88 | b | **SBv** |
| WX-A_GROUP//WX-A-RC222 | TT_CC | 759.9 | 487.4 | c | **SBv** |
| SBIIb | TT | 6.65 | 0.55 | a | **GT-ASV** |
| SBIIb | CC | 3.64 | 1 | b | **GT-ASV** |

**Supplemental Table 6**. **Grain quality Total and Additive genetic trend table.**

| Trait | Total  baseline | Total Trend | Total Std. Error | Total Slope | Total *P*-value | Additive baseline | Additive trend | Additive Std. Error | Additive Slope | Additive P-value |
| --- | --- | --- | --- | --- | --- | --- | --- | --- | --- | --- |
| *OHRR* | 60.48 | -0.1006 | 0.040 | -0.1663 | 0.0122* | 60.24 | -0.1 | 0.05 | -0.16 | 0.05* |
| *DHRR* | 40.91 | 0.3833 | 0.100 | 0.9370 | 1x10^-4** | 43.62 | 0.03 | 0.13 | 0.06 | 0.82 |
| *AC* | 29.81 | 0.0073 | 0.025 | 0.0245 | 0.7721 | 29.53 | 0.041 | 0.034 | 0.14 | 0.2 |
| *GT-ASV* | 5.91 | 0.0256 | 0.017 | 0.4332 | 0.1389 | 5.79 | 0.04 | 0.022 | 0.68 | 0.07 |
| *BDv* | 852.64 | -2.0716 | 3.339 | -0.2430 | 0.535 | 900.2 | -8.24 | 4.28 | -1 | 0.05* |
| *SDv* | 1289.95 | 7.6986 | 6.426 | 0.5968 | 0.2309 | 1147 | 24.59 | 8.29 | 2.15 | 0.003** |
| *GL* | 6.90 | 0.0145 | 0.004 | 0.2103 | 2x10^-4** | 6.95 | 0.008 | 0.005 | 0.1 | 0.11 |
| *GW* | 2.24 | -0.0049 | 0.001 | -0.2187 | 6.6x10^-6** | 2.23 | -0.003 | 0.001 | -0.16 | 0.01** |
| *GLWR* | 3.08 | 0.0141 | 0.003 | 0.4573 | 3.7x10^-7** | 3.14 | 0.006 | 0.003 | 0.2 | 0.06 |
| *WCRK* | 11.37 | -0.273 | 0.065 | -2.4 | 2.7x10^-5** | 9.09 | -0.11 | 0.088 | -1.1 | 0.17 |
